# Identification of western North Atlantic odontocete echolocation click types using machine learning and spatiotemporal correlates

**DOI:** 10.1371/journal.pone.0264988

**Published:** 2022-03-24

**Authors:** Rebecca E. Cohen, Kaitlin E. Frasier, Simone Baumann-Pickering, Sean M. Wiggins, Macey A. Rafter, Lauren M. Baggett, John A. Hildebrand

**Affiliations:** Scripps Institution of Oceanography, University of California San Diego, La Jolla, California, United States of America; Wildlife Conservation Society Canada, CANADA

## Abstract

A combination of machine learning and expert analyst review was used to detect odontocete echolocation clicks, identify dominant click types, and classify clicks in 32 years of acoustic data collected at 11 autonomous monitoring sites in the western North Atlantic between 2016 and 2019. Previously-described click types for eight known odontocete species or genera were identified in this data set: Blainville’s beaked whales (*Mesoplodon densirostris*), Cuvier’s beaked whales (*Ziphius cavirostris*), Gervais’ beaked whales (*Mesoplodon europaeus*), Sowerby’s beaked whales (*Mesoplodon bidens*), and True’s beaked whales (*Mesoplodon mirus*), *Kogia spp*., Risso’s dolphin (*Grampus griseus*), and sperm whales (*Physeter macrocephalus*). Six novel delphinid echolocation click types were identified and named according to their median peak frequencies. Consideration of the spatiotemporal distribution of these unidentified click types, and comparison to historical sighting data, enabled assignment of the probable species identity to three of the six types, and group identity to a fourth type. UD36, UD26, and UD28 were attributed to Risso’s dolphin (*G*. *griseus*), short-finned pilot whale (*G*. *macrorhynchus*), and short-beaked common dolphin (*D*. *delphis*), respectively, based on similar regional distributions and seasonal presence patterns. UD19 was attributed to one or more species in the subfamily *Globicephalinae* based on spectral content and signal timing. UD47 and UD38 represent distinct types for which no clear spatiotemporal match was apparent. This approach leveraged the power of big acoustic and big visual data to add to the catalog of known species-specific acoustic signals and yield new inferences about odontocete spatiotemporal distribution patterns. The tools and call types described here can be used for efficient analysis of other existing and future passive acoustic data sets from this region.

## Introduction

Odontocetes, or toothed whales, are vocal species which use sound for social communication, foraging, and navigation [1, 2], making them prime targets for passive acoustic monitoring. Although the acoustic repertoire of some odontocete species have been well-studied, many remain understudied, have yet to be acoustically characterized, and cannot be distinguished to the species level in passive acoustic recordings. This limits the utilization of passive acoustic data sets for odontocete population assessments and ecological studies. Echolocation clicks, short in duration and mostly broadband biosonar impulses, are produced by odontocete species across a range of behavioral contexts. Species-specific echolocation click types, exhibiting characteristic spectral and temporal features, have been discovered for a broad range of odontocete species including sperm whales [3], *Kogia spp*. [4], beaked whales [5], and Risso’s dolphins [6]. Discriminating features typically include spectral peaks and/or notches, and clicking rate. The challenge of identifying robust patterns within naturally variable signals is considerable when working with echolocation clicks due to the substantial signal variability observed in response to both environmental conditions and behavioral state [7–12]. Additionally, these signals are abundant—tens of millions of clicks from a dozen or more species can easily be recorded over the course of a year of recording effort at a monitoring site—making them good candidates for automated signal discovery.

The analysis of marine acoustic data has traditionally been highly labor intensive, with expert analysts manually logging individual encounters, calls, or even clicks of their target species. Such approaches are severely limited by the rate at which the data can be thus analyzed, and in recent years the rate and volume of passive acoustic data collection has outstripped the pace of manual analysis. These expert analyst methods are also less than ideal in terms of reproducibility and objectivity [13]. Over the past decade the development of machine learning tools, and their applications to ecological data, has resulted in a proliferation of automated methods for analyzing large marine acoustic datasets. Both unsupervised and supervised learning frameworks, most notably clustering and deep learning algorithms, have become standard tools in the analysis of marine acoustic data [14–24]. These approaches require initial time investment to develop the models, and for some applications this investment may be substantial. For example, the creation of labeled training and testing sets for supervised learning is a notably time- and labor-intensive process. But once a model has been adequately trained, analysis of large datasets can be readily accomplished. Automated signal discovery, detection, and classification algorithms (with the latter two sometimes occurring in a single step) have demonstrated good success for a number of marine mammal species [see, e.g., Refs 15, 16, 18, 19, 22–25] with improved objectivity and reproducibility compared to manual analysis.

As underwater autonomous passive acoustic recordings are collected without associated species presence metadata, species-level attribution of novel signals found in these data require drawing on other data sources. Traditional marine mammal line-transect visual surveys provide high confidence species presence and group size data, and in some regions such surveys have been carried out regularly for years or even decades. These two modalities are highly complementary, with passive acoustic devices providing high temporal resolution long-term time series of acoustic presence at discrete sampling points, and visual surveys providing snapshots of animal presence over survey track lines or grids. When aggregated over years and across sites, both can give an indication of long-term species range and distribution patterns over large regions. By combining these disparate data streams, it may be possible to gain insights about which species are producing a newly identified acoustic signal type. Simultaneous passive acoustic recordings and visual sightings can be especially valuable as such instances may provide explicit labels for encounters within the acoustic data set.

In this work, we identify novel species-specific odontocete echolocation click types, and determine the species most likely responsible for producing them. This was accomplished using an unsupervised signal discovery and labeling approach on a large autonomous passive acoustic data set from the U.S. eastern seaboard. By combining machine learning techniques with expert analyst review, we identified recurring signal types that exhibited the characteristics of odontocete echolocation clicks. The spatiotemporal distribution patterns exhibited by these signals were compared to the historical distribution of sighting data for each odontocete species known to be present in the region to correlate species presence with click types. Opportunistic encounters captured in both the acoustic and the historical sighting data were used where available to build evidence for species attributions. This approach yielded six novel delphinid click types; likely species assignments were identified for three types (UD36, UD26, and UD28), and a group-level assignment was identified for a fourth type (UD19), with two click types remaining unidentified. These novel species assignments will enable further study of these species’ spatiotemporal distribution patterns and ecology using passive acoustic recordings collected in this region, and may be an indication of the signals attributable to the same species in other regions.

## Methods

### Data collection

**Fig 1:**
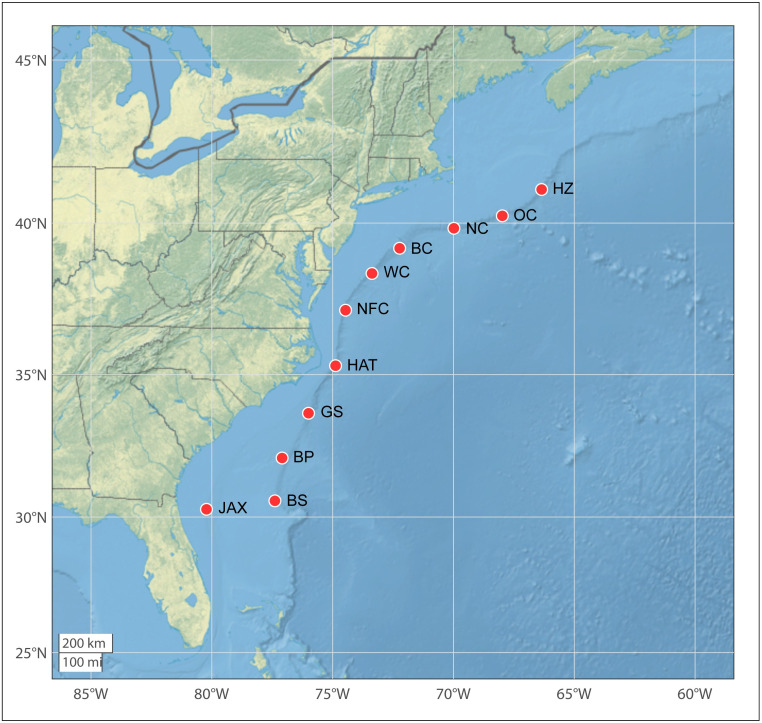
Western North Atlantic study area with long-term autonomous passive acoustic monitoring sites (red circles) and associated site name abbreviations. HZ = Heezen Canyon; OC = Oceanographer’s Canyon; NC = Nantucket Canyon; BC = Babylon Canyon; WC = Wilmington Canyon; NFC = Norfolk Canyon; HAT = Hatteras; GS = Gulf Stream; BP = Blake Plateau; BS = Blake Spur; JAX = Jacksonville.

Passive acoustic data were collected using High-frequency Acoustic Recording Packages (HARPs) [26] deployed at 11 continental shelf break and slope sites between 30° N and 42° N in the western North Atlantic (Fig 1). The devices were deployed at depths of approximately 450 m to 1,350 m and recorded continuously with a sampling rate of 200 kHz and 16-bit analog-to-digital conversion. Most devices were equipped with a single omnidirectional sensor (International Transducer Corporation’s ITC-1042). Seven deployments used devices with separate low frequency (Teledyne Benthos AQ-1) and high-frequency (ITC1042) sensors. Both devices had well-characterized combined frequency response between 10 Hz and 100 kHz. A bandpass filter reduced low-frequency noise and high-frequency aliasing. Devices recorded for between 4 months and 14.5 months per deployment; repeated redeployments at each site enabled almost uninterrupted recording from Spring 2016 to Spring 2019 (Table 1), totaling just over 32 years of recording effort across sites.

**Table 1:** HARP deployment information for repeated deployments at the acoustic monitoring sites shown in Fig 1.

Site	Deployment	Latitude	Longitude	Depth (m)	Data Start Date & Time		Data End Date & Time	
Heezen Canyon (HZ)	1	41°03.71’ N	66°21.10’ W	883	4/22/2016	18:00:00	6/19/2017	7:05:06
	2	41°03.70’ N	66°21.09’ W	885	7/9/2017	0:00:00	1/13/2018	15:25:06
	3	41°03.70’ N	66°21.09’ W	885	6/11/2018	17:59:59	5/10/2019	6:33:44
Oceanographer’s Canyon (OC)	1	40°15.80’ N	67°59.17’ W	448	4/24/2016	5:59:59	5/18/2017	6:37:35
	2	40°15.80’ N	67°59.18’ W	447	7/6/2017	23:59:59	4/16/2018	5:56:18
	3	40°13.80’ N	67°58.68’ W	882	6/10/2018	6:00:00	5/19/2019	4:33:45
Nantucket Canyon (NC)	1	39°49.94’ N	69°58.93’ W	894	4/21/2016	18:00:00	5/24/2017	14:53:51
	2	39°49.96’ N	69°58.92’ W	894	7/16/2017	18:00:00	6/9/2018	13:02:36
	3	39°49.98’ N	69°58.92’ W	894	6/10/2018	0:00:00	6/3/2019	4:43:45
Babylon Canyon (BC)	1	39°11.46’ N	72°13.72’ W	999	4/20/2016	18:00:00	6/10/2017	23:04:05
	2	39°11.43’ N	72°13.63’ W	1003	6/30/2017	12:00:00	6/3/2018	11:31:21
	3	39°11.52’ N	72°13.64’ W	997	6/3/2018	12:00:00	5/19/2019	19:30:00
Wilmington Canyon (WC)	1	38°22.45’ N	73°22.24’ W	1028	4/20/2016	6:00:00	6/29/2017	20:57:36
	2	38°22.43’ N	73°22.21’ W	1036	6/30/2017	0:00:00	6/2/2018	20:42:36
	3	38°22.40’ N	73°22.19’ W	1045	6/2/2018	22:00:00	5/19/2019	8:32:30
Norfolk Canyon (NFC)	1	37°09.99’ N	74°20.00’ W	1028	4/30/2016	12:00:00	6/28/2017	18:38:51
	2	37°10.04’ N	74°27.98’ W	992	6/30/2017	0:00:00	6/2/2018	16:15:06
	3	37°09.87’ N	74°27.95’ W	1111	6/2/2018	12:00:00	5/18/2019	17:46:40
Hatteras (HAT)	1	35°18.11’ N	74°52.74’ W	1194	4/29/2016	12:00:00	2/6/2017	8:56:03
	2	35°35.05’ N	74°44.99’ W	1128	5/9/2017	12:02:54	10/25/2017	14:11:45
	3	35°35.01’ N	74°44.58’ W	1222	10/26/2017	12:00:00	6/1/2018	0:54:59
	4	35°35.39’ N	74°44.86’ W	1327	6/1/2018	4:00:00	12/14/2018	14:42:36
	5	35°35.36’ N	74°45.27’ W	1208	12/14/2018	0:00:00	5/17/2019	18:17:30
Gulf Stream (GS)	1	33°39.94’ N	76°00.08’ W	926	4/29/2016	0:00:00	6/27/2017	18:35:06
	2	33°40.02’ N	75°59.97’ W	932	6/28/2017	0:00:00	6/26/2018	11:31:21
	3	33°40.20’ N	75°59.86’ W	933	6/28/2018	23:59:59	6/18/2019	14:17:09
Blake Plateau (BP)	1	32°06.36’ N	77°05.66’ W	953	4/28/2016	12:00:00	6/27/2017	4:57:36
	2	32°06.42’ N	77°05.41’ W	951	6/27/2017	12:00:00	6/28/2018	13:08:51
	3	32°06.32’ N	77°05.44’ W	950	6/28/2018	0:00:00	5/28/2019	4:01:15
Blake Spur (BS)	1	30°35.03’ N	77°23.44’ W	1047	4/27/2016	18:00:00	6/26/2017	15:22:05
	2	30°34.98’ N	77°23.43’ W	1047	6/26/2017	18:00:00	6/23/2018	7:32:33
	3	30°34.98’ N	77°23.40’ W	1047	6/28/2018	0:00:00	6/16/2019	20:13:45
Jacksonville (JAX)	1	30°09.11’ N	79°46.21’ W	748	4/26/2016	18:00:00	6/25/2017	19:23:35
	2	30°09.16’ N	79°46.19’ W	748	6/25/2017	18:03:57	10/28/2017	17:27:48
	3	30°09.14’ N	79°46.24’ W	746	6/27/2018	0:00:00	6/15/2019	11:03:45

Publicly available historical visual survey data were accessed on Duke University’s OBIS-SEAMAP database [27] (individual data set citations are found in the S2 Text) to compile a record of odontocete species sightings in the western North Atlantic. A total of 58,320 sightings were compiled for 26 odontocete species, ~52% from shipboard surveys, ~40% from aerial surveys, and the remainder from shore stations. The geographical limits of 63°–82° W and 24°–46° N were chosen to bound the study area, and sightings outside these limits were excluded. Rare data from as far back as 1913 were included in the analysis, but ~94% of the sightings occurred during 1980–2019.

### Signal detection & classification

All analyses were carried out in MATLAB (Mathworks, Inc., Natick, MA, USA) using custom routines and a combination of automated methods and manual analysis developed by Frasier et al. [14, 18, 28]. This combined approach enables efficient signal detection, signal type discovery, and classification of large numbers of echolocation clicks with consistent, objective criteria, while simultaneously incorporating analyst review to ensure the resultant detections, signal types, and classifications are meaningful and not simply artifacts of the automated algorithms.

Echolocation clicks were identified using a 2-step automated detection routine [28]. In the first step, acoustic data were filtered with a 5-pole Butterworth filter with a passband between 5 kHz and 100 kHz, and then waveform samples exceeding a peak-to-peak threshold of 118 dB re:1 μPa were identified; areas of interest were expanded to include all samples within 2.5 ms of each high amplitude peak, and high-amplitude events separated by <2.5 ms were merged. In the second step, individual impulsive signals were located within these high-amplitude events by identifying samples exceeding the amplitude threshold; individual signal start and end times were then defined as the first and last sample on either side of the main peak which exceeded the 70th percentile of energy for the entire high-energy event. Individual signals separated by <100 μs were merged, and clipped signals were discarded. Descriptive parameters were calculated for each candidate click (duration, spectrum (400-point FFT yielding a 500 Hz spectral resolution, Hann window, 50% overlap), peak frequency, peak-to-peak amplitude at the peak frequency, -3dB bandwidth, and envelope shape) and compared to user-defined thresholds to determine whether to retain or discard the impulse. The goal was to capture as many odontocete echolocation clicks as possible, particularly previously undescribed types; therefore, thresholds were set to span the range of variability of known odontocete click types based on previous works [3–6, 29–34]. This approach was anticipated to also capture many non-click signals, which would be classified as non-target events in the subsequent steps. The detector was run on each deployment independently, yielding time series of putative clicks and their parameters from each site. Detected clicks in this analysis were not evaluated to identify on-axis arrivals, but rather all detected clicks were retained for the clustering and classification steps, to identify dominant signal types across detections.

To identify dominant click types at each site the unsupervised clustering approach developed by Frasier *et al*. [18], also a 2-step process, was used to cluster each deployment independently. Identical settings were used to cluster all deployments to allow direct comparison of the final clusters across deployments. In the first step, deployments were divided into 5-minute time bins and the Chinese whispers algorithm [35] was used to cluster detections in each bin based on pairwise spectral distances; only clicks with peak-to-peak sound pressure levels ≧120 dB re:1μPa were clustered. 5-minute bin durations were selected as a trade-off between maintaining high temporal resolution while considering a time period within which there were likely to be sufficient clicks for the clustering algorithm to identify meaningful groupings, and simultaneously reducing the large volume of data of each deployment down to a more tractable size for the second clustering step. This determination was made based upon the slowest odontocete clicking rate in our analysis, that of sperm whales, which may click as slowly as once per second [36], and the expectation that some clicks would have been excluded by the detector during low-amplitude encounters (distant animals). Additionally, this bin duration was considered short enough to capture possible evolutions in click characteristics over the course of a given encounter (typically tens of minutes to several hours). An edge pruning parameter *p*_*e*_ = 0.95 was used for the first clustering iteration, consistent with the approach of Frasier *et al*. [18] resulting in the formation of on average 1.2 clusters per bin. Multiple clusters formed in a bin if there were sufficient clicks representing two or more distinctly different signal types. Mean spectrum, inter-click-interval (ICI) distribution, and mean waveform envelope were calculated for each cluster formed in each 5-minute bin. In the second step, the same algorithm was used to cluster a subsample of 40,000 bin-level spectra per deployment by comparing spectral shape as well as mean waveform envelope. This step was memory-limited, and selecting a subset of the bin-level averages was necessary due to the computational demands of the clustering algorithm. To improve the robustness of the clusters formed by this second step, clusters consisting of fewer than 25 bin-level averages representing a minimum of 50 individual detections each were discarded. These requirements reduced the formation of clusters based on short-lived noise events, or a small number of randomly similar noises, but likely also resulted in rare click types not being represented in the final clusters. A pruning parameter *p*_*e*_ = 0.98 was selected for this step by comparing several clustering iterations run with varying parameter values (0.95 ≤ *p*_*e*_ ≤ 0.99) and considering cluster consistency versus unnecessary separation of highly similar clusters. As with the detector, consistent settings were used for all deployments to allow meaningful comparison of the clusters formed across deployments. Mean summary spectra per cluster, ICI distributions, concatenations of contributing bin-level spectra, and concatenations of contributing bin-level mean waveform envelopes, along with information about which bin-level spectra contributed to each cluster, were saved for the output from this step.

Clusters arising from this second step were manually compared across sites to identify recurring signal types. Clusters were compared on spectral shape and ICI distribution, with consideration given to the self-similarity of a cluster (i.e., the consistency of apparent spectral features across all contributing bins, an indication of cluster quality), the number of bins contributing to each cluster, the number of sites an apparent type was present at, and the consistency of an apparent type at those sites across the three-year study period. Multiple clusters from a given site were allowed to contribute to an apparent type, on the premise that click types show substantial natural variability and the stringency of the clustering process may have led to overzealous cluster separation. Eighteen (18) distinct recurring impulse types were identified, each of which was classified as either: 1) a previously-described click type attributable to a known species; 2) a recurrent signal which appeared to be an odontocete click type, but whose species of origin was unknown; or 3) a non-odontocete impulse from a noise source such as anthropogenic sonar or cavitation bubbles. Sonar was easily identified by the concentration of energy in narrow spectral bands, long-duration signal envelopes compared to echolocation clicks, and multi-modal inter-signal-interval histograms which arose from pooling data from sonar operating with different ping rates. Differentiation between odontocete click types and cavitation bubbles (e.g. ship propellers, snapping shrimp) was based largely on signal timing, relying on the tendency of odontocetes to produce click trains with fairly regular and species-specific timing [5, 37], while cavitation bubbles are produced at random. Descriptive parameters (mean power spectrum, peak frequencies, 3dB bandwidth) were calculated for each type based on 2,000 representative clicks. The ICI median of modes for each type based on the ICI distributions from 1,000 5-minute bins containing clicks from that type was computed. In the modal ICI distribution plots below, values <0.02 s have been suppressed to reduce the contribution of high density encounters in which ICIs values are saturated with near-zero values due to the interleaving of click trains from many individuals clicking simultaneously.

The final 18 types selected from the clustering process, as well as a class representing Gulf of Mexico Gervais’ beaked whales [38] and another class from the same Gulf of Mexico data representing snapping shrimp [39], which have been previously observed in acoustic data from the JAX site, were used to establish training classes for a deep neural network-based classifier. We hypothesized that the Gulf of Mexico Gervais’ population, which may or may not migrate between the Gulf of Mexico and the Atlantic, might be distinct and acoustically identifiable; therefore, Gulf of Mexico Gervais’ clicks were included as a separate class to test whether their presence was detected at the Atlantic monitoring sites. Five noise classes accounting for several of the common noise types were included so that these signals would not end up incorrectly labeled as odontocete clicks for lack of an outgroup: ship noise, snapping shrimp, and 3 classes of sonar separated by frequency content. Another important consideration was the maximum number of classes a classifier can be realistically expected to discriminate between with an acceptable level of error, as the likelihood of correct classification is inversely proportional to the number of classes.

Several different types of training data and neural network architectures were tested to tune the hyperparameters of the model and optimize performance. Examples for each class were either subsampled (for well-represented classes) or augmented via simulation (for minority classes) to obtain a balanced set of 5,500 examples per class. Augmentation was carried out by adding Gaussian noise to existing examples, resulting in new examples which retained the defining characteristics of their target classes while avoiding redundancy. Examples were randomly subdivided for training (5000 examples) and testing (500 examples); training data were further randomly subdivided for training and validation using an 80/20 split: 80% for training and 20% for validating performance. Final network architecture consisted of four 512-node fully connected layers with rectified linear unit (ReLU) [40] activation, 50% dropout between fully connected layers, batch normalization after the last two dropout layers, and a softmax [41] output layer. Highest test accuracy was attained by training on spectral shape, click rate, and waveform envelope shape (S1 Table).

The trained model was run on all HARP deployments, yielding labels and associated probabilities for each 5-minute bin-level mean spectrum. Classifier performance on novel data was expected to have different accuracy than that which was achieved on the training and testing sets due to the occurrence of intermediate and noisy clicks and signal types which do not belong to any of the available classes. To account for this, a high-level review of the bin-level labels for each deployment was carried out to remove obviously incorrect labels. For each deployment, spectra assigned to each class were sorted by peak frequency and concatenated for visual comparison; spectra whose frequency content was highly inconsistent with the characteristics of their labeled class were manually flagged for removal. This step was carried out conservatively to remove blatantly incorrect labels while leaving untouched both good and questionable labels, in hopes of retaining all bins which seemed to possibly indicate presence for each class. Residual classifier error was then estimated by calculating the false positive rate (FPR) for a stratified random subset of the retained labels. This approach was favored over the quantification of confusion due to the uncertainty involved in the assignment of noisy and intermediate clicks to a “true” class. The effects of enforcing increasingly high received level and number-clicks-per-bin thresholds were explored as approaches to minimize FPR by attempting to exclude poor quality clicks.

### Spatiotemporal correlation

To assess regional and temporal patterns in the distribution of the click types, average seasonal acoustic presence of respective click types across acoustic monitoring site were plotted as scaled bubble maps. Hours of acoustic presence were first summed within each season and normalized by recording effort to account for gaps between deployments, and then seasons were averaged across the three-year study period. Seasons were defined as: Spring: March-May; Summer: June-August; Fall: September-November; Winter: December-February. Classifier error (FPR, averaged across repeated deployments at each site) was used to scale bubbles to avoid misleading bubble sizes at sites where error was high for a given click type.

Maps of historical sighting data were similarly plotted for each odontocete species (the exception being the two *Kogia* species, *breviceps and sima*, which were grouped by genus as *Kogia spp*. due to the challenges of discriminating between these species at sea) to allow direct comparison to the click type bubble maps. Seasonal sightings were pooled for each season across all years of data for each species, rather than averaged, due to large interannual differences in survey effort and sighting rates. Survey track lines traveled in each season were plotted when available (138 of 197 total datasets), to give a sense of where lack of sightings may be confounded by lack of visual survey effort.

To further support species-specific click type identifications based on matches between acoustic and visual distributions and seasonal patterns, delphinid sightings occurring within the estimated recording radius (~2 km) [42] of the acoustic mooring sites were identified and the acoustic data collected during these known species encounters was examined to identify associated echolocation events. Sightings recorded within close proximity of any acoustic mooring were rare due to a lack of coordinated visual and acoustic monitoring effort, but 4 qualifying encounters were identified.

## Results

Of the 20 classes established based on the clustering output and used to train the neural network, nine represented known odontocete species or genera: Blainville’s beaked whale (*M*. *densirostris*), Cuvier’s beaked whale (*Z*. *cavirostris*), Atlantic Gervais’ beaked whale (*M*. *europaeus*), Gulf of Mexico Gervais’ beaked whale (*M*. *europaeus*), Sowerby’s beaked whale (*M*. *bidens*), True’s beaked whale (*M*. *mirus*), *Kogia spp*., Risso’s dolphin (*G*. *griseus*), sperm whale (*P*. *macrocephalus*). Six appeared to be delphinid clicks whose species of origin were unknown. The remaining five classes represented a variety of noise sources which were included in the classifier to reduce the incidence of false positives: snapping shrimp, ship cavitation, high-, mid-, and multi-frequency sonar. The unidentified click types were presumed to be generated by delphinids and not beaked whales based on their waveforms with few oscillations, and short, delphinid-like ICIs [10, 43–45]. These delphinid click types were named “UD” for “unidentified delphinid,” followed by the approximate value of the median peak frequency in kHz (e.g. “UD36”). They can be differentiated by their signal parameters peak frequency, 3dB bandwidth, and modal ICI (Table 2). An overview of the results for the noise classes is available in the S1 Text, S1 Fig.

**Table 2:** Signal parameters peak frequency, 3dB bandwidth, and modal ICI for known species and novel click types given as median with 10th and 90th percentile in brackets. Names for known-type classes are abbreviations of the species/genus names: Gg: *Grampus griseus*; Mb: *Mesoplodon bidens*; Md: *Mesoplodon densirostris*; Me: *Mesoplodon europaeus*; Mm: *Mesoplodon mirus*; Zc: *Ziphius cavirostris*; Kogia: *Kogia spp*.; Pm: *Physeter macrocephalus*.

Click Type	Peak Frequency (kHz)	3dB Bandwidth (kHz)	Modal ICI (s)
Gg	32.5 [23.5,38.0]	4.5 [2,10]	0.145 [0.085,0.195]
Mb	67.0 [59.5,73.5]	13.5 [6.5,21.0]	0.135 [0.125,0.185]
Md	31.5 [28.5,35.5]	7.5 [3,11]	0.325 [0.225,0.375]
Me	46.5 [38.0,74.5]	11.5 [4.5,20.5]	0.285 [0.245,0.305]
Mm	47.5 [41.0,75.0]	11.3 [3.50,21.5]	0.185 [0.165,0.205]
Zc	38.5 [31.0,42.5]	7.0 [3.5,13.5]	0.465 [0.085,0.535]
Kogia	99.5 [93.0,99.5]	7.0 [4.0,12.5]	0.085 [0.065,0.115]
Pm	8.5 [6.5,13.0]	3.0 [1.5,5.5]	0.475 [0.035,0.655]
UD36	36.5 [30.0,47.0]	5.5 [2.5,13.0]	0.155 [0.135,0.177]
UD26	26.5 [12.0,39.0]	4 [2.0,9.5]	0.165 [0.085,0.195]
UD28	28.5 [23.0,34.3]	9 [3.0,15.0]	0.075 [0.045,0.105]
UD19	19.0 [15.0,26.5]	9 [3.5,15.0]	0.135 [0.035,0.225]
UD47	47.0 [19.5,57.0]	6.5 [2.5,15.5]	0.065 [0.055,0.085]
UD38	38.5 [29.3,46.5]	8 [4.0,14.5]	0.065 [0.055,0.085]

All odontocete click types exhibited distinct regional and seasonal patterns in distribution and acoustic density. Absolute magnitude of acoustic presence, in terms of average seasonal hours per site (scaled by FPR), varied substantially between click types. UD28 exhibited a maximum presence at NFC each spring, averaging 901 hours, while *Kogia* presence peaked at an average of 9.5 hours at GS in the winter.

### Risso’s dolphin (*Grampus griseus*)

#### Description

**Fig 2:**
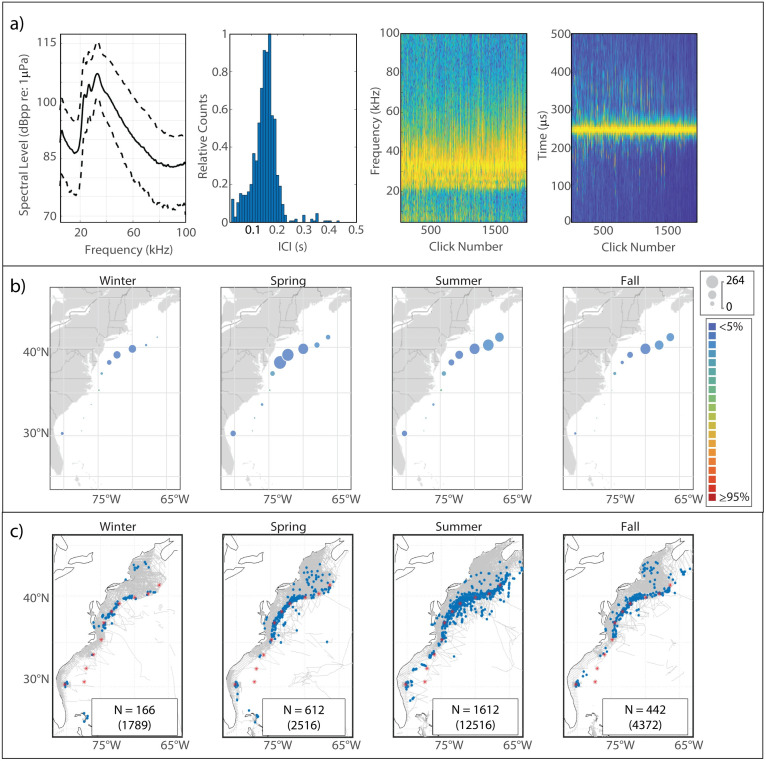
Results for Risso’s dolphin showing click type (a), acoustic presence (b), and historical sightings (c). Click type plots, from left to right: median power spectrum (solid line) with 10^th^ and 90^th^ percentiles (dashed lines); distribution of modal ICI values from 1,000 5-minute bins; concatenation of normalized click spectra, sorted by received level; concatenation of normalized waveform envelopes, sorted by received level. For the concatenated spectra and waveform envelopes, the normalized magnitude of the frequency/pressure is represented by color such that warmer colors show greater magnitude. Acoustic presence shown as scaled circles depicting cumulative hours at each acoustic monitoring site per season, averaged across three years of data; classifier error given by color per legend in (b). Historical sightings per season (blue dots), shown relative to acoustic monitoring sites (red stars) and track lines of surveys undertaken in each season (grey lines). Inset within each sighting map shows number of sightings (N); total number of individuals summed across all sightings for which group size data was available is shown in parentheses.

This click type, known to be generated by Risso’s dolphins based on previous works [6, 10], is characterized by a multi-peaked structure (Fig 2a). The Risso’s clicks in our analysis exhibited lower-amplitude peaks at 23.5 kHz and 27 kHz, and a narrow main peak reaching maximum amplitude at a median frequency of 33 kHz. The modal ICI value of 0.145 s was on the longer side for delphinids, and was consistent with the relatively large body size of Risso’s dolphins.

#### Spatiotemporal distribution

The Risso’s click type showed a predominantly northerly distribution, although it was present at every acoustic monitoring site in every season (Fig 2b). A clear seasonal pattern was visible, with highest presence at WC, BC, and NC in the spring shifting northward to highest presence at NC, OC, and HZ in the summer and into the fall; winter presence was lower at all of the northern sites. JAX exhibited the highest levels of acoustic presence of the southern sites, with a distinct maximum in the spring and summer and minimum in the fall and winter. Historical sightings of Risso’s dolphins map quite well to the acoustic presence of the Risso’s click type (Fig 2c).

### Sowerby’s beaked whale (*Mesoplodon bidens*)

#### Description

**Fig 3:**
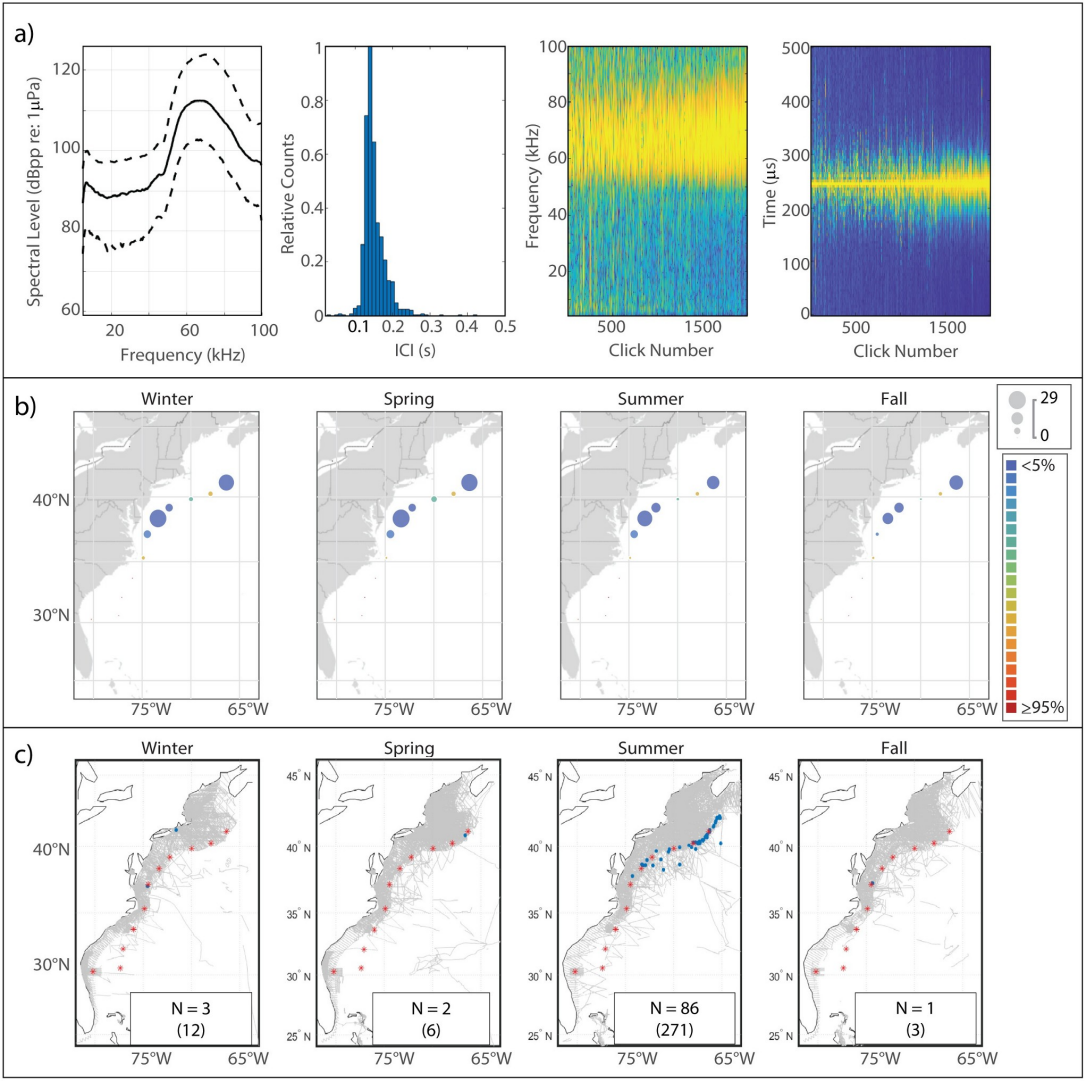
Results for Sowerby’s beaked whale showing click type (a), acoustic presence (b), and historical sightings (c). Subplots as in Fig 2.

Sowerby’s beaked whales produce clicks with energy distributed across a wide band from 50 kHz to 90 kHz [31, 46] (Fig 3a). We found the median peak frequency to be 67 kHz. The median modal ICI value, 0.135 s, was surprisingly short for a large-bodied species, but was consistent with previous findings.

#### Spatiotemporal distribution

Overall acoustic presence of Sowerby’s was quite low at our monitoring sites, but an interesting distribution pattern was visible with maxima in presence in two distinct regions—one in the WC area and another further north at HZ (Fig 2b). Highest levels of presence in both regions were seen in the spring, while presence was lowest in the fall, although the amplitude of this seasonal fluctuation was not very large. Sightings of Sowerby’s beaked whales were rare, and most commonly occurred near the shelf break of Georges Bank in the summer; this pattern was not mirrored in the acoustic presence, although the pattern of a northerly distribution is visible in both sets of maps.

### Blainville’s beaked whale (*Mesoplodon densirostris*)

#### Description

**Fig 4:**
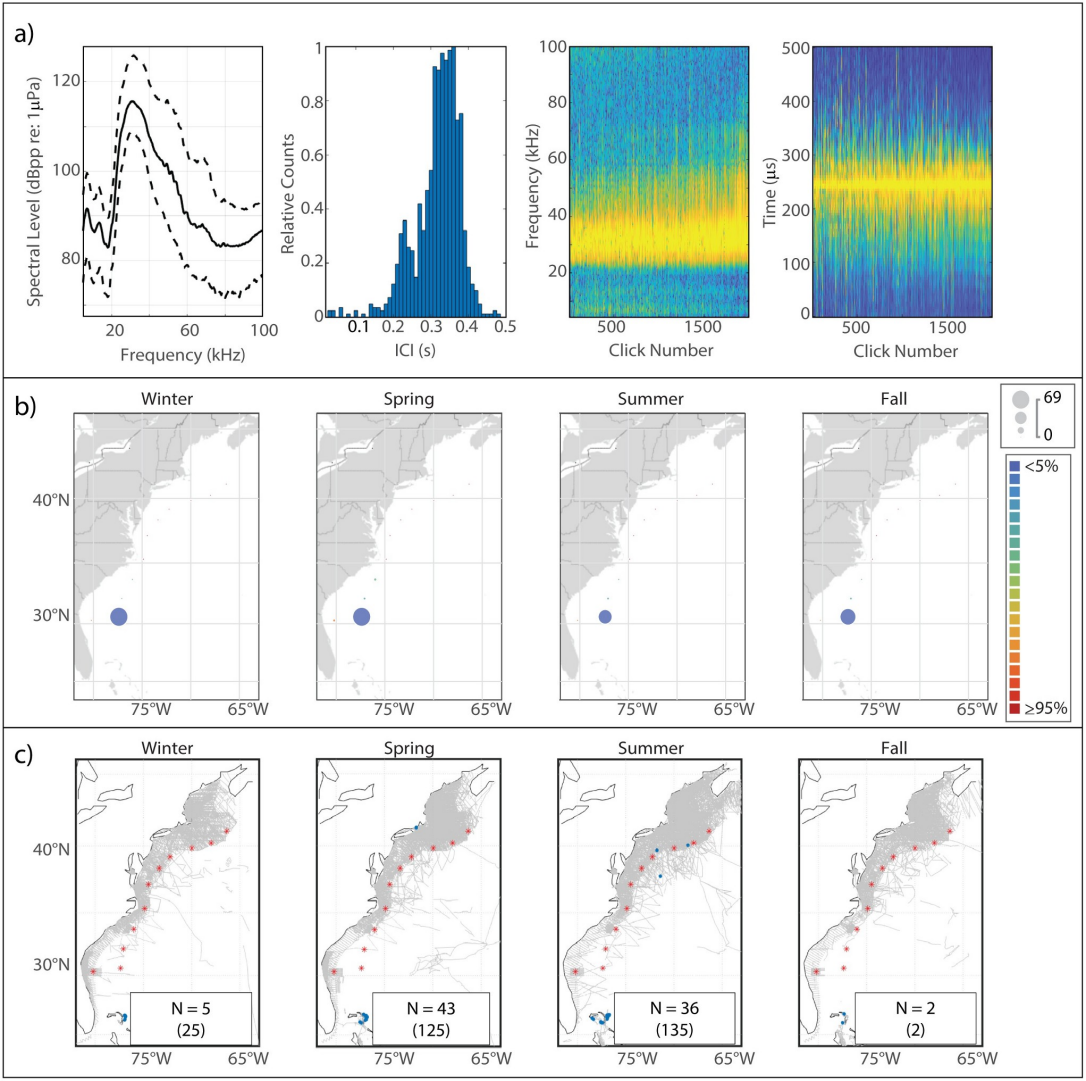
Results for Blainville’s beaked whale showing click type (a), acoustic presence (b), and historical sightings (c). Subplots as in Fig 2.

This click type, known to be attributable to Blainville’s beaked whale [5, 47], exhibited a sharp onset of energy around 25 kHz and a single peak which, in our analysis, attained highest amplitude at a median frequency of 31.5 kHz (Fig 4a). The median modal ICI value was 0.325 s.

#### Spatiotemporal distribution

Blainville’s exhibited the greatest acoustic presence at BS, where a slight summer decline in presence was visible (Fig 4b). Presence was negligible across the other monitoring sites, but a very slight increase at GS, BP, and JAX was visible in the spring. Sightings of Blainville’s were rare and occurred mostly near the Bahamas (Fig 4c).

### Gervais’ beaked whale (*Mesoplodon europaeus*)

Very few clicks were classified as Gulf of Mexico Gervais’ (maximum 1.9 hours at JAX in spring), and the clicks classified as Atlantic Gervais did not appear meaningfully different from those classified as Gulf of Mexico Gervais’. Therefore we concluded that these types are not currently differentiable using our methods, and have combined the two classes here. It remains unclear whether this is because there is no acoustic distinction to be made between the two, or because the Gulf of Mexico whales do not migrate to the Atlantic, providing no true Gulf of Mexico Gervais’ encounters for the classifier to identify.

#### Description

**Fig 5:**
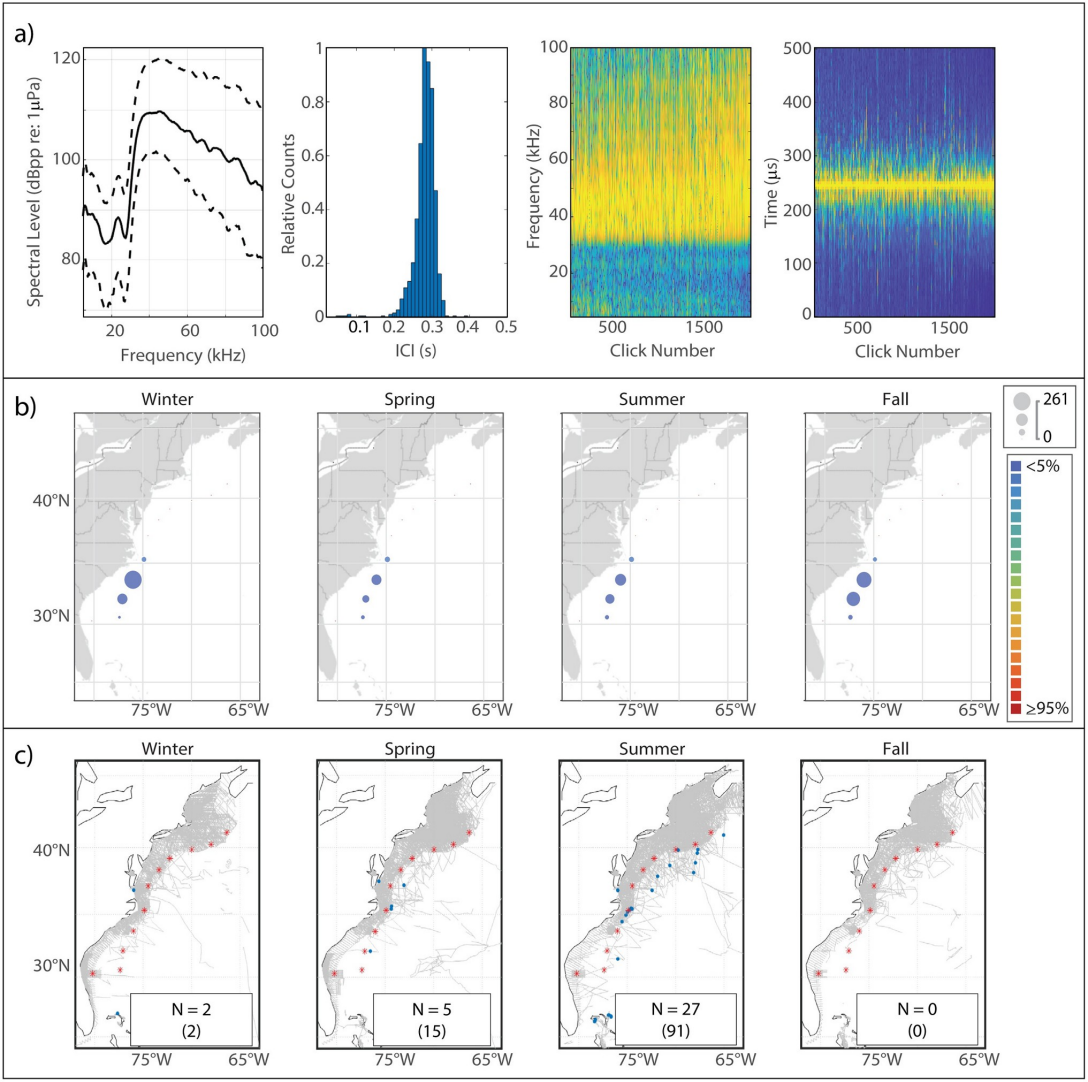
Results for Gervais’ beaked whale showing click type (a), acoustic presence (b), and historical sightings (c). Subplots as in Fig 2.

The type attributed to Gervais’ beaked whale was characterized by a sharp onset of energy at around 30 kHz [48] (Fig 5a). The Gervais’ clicks in our analysis reached peak amplitude at a median frequency of 46.5 kHz, with a much lower amplitude peak present at 23.5 kHz. We observed that the rate of energy drop-off above 50 kHz seemed to be a function of received level, with higher amplitude clicks exhibiting only a small diminishment in amplitude at the higher frequencies, and lower-amplitude clicks exhibiting a much steeper rate of drop-off. The median modal ICI value was 0.275 s.

#### Spatiotemporal distribution

The acoustic presence of the Gervais’ click type lived up to this species’ moniker of “Gulf Stream beaked whale”, with highest presence at the Gulf Stream monitoring site (Fig 5b). Overall distribution was strictly southerly and mostly focused at the GS and BP sites, with lower levels of presence at HAT and BS and no presence at JAX. There was a distinct seasonal pattern apparent, with an increase in presence at GS and BP beginning in the fall and reaching a maximum in the winter, and lower levels of presence in the spring and summer. Sightings of Gervais’ beaked whales were very rare, with just 34 sightings reported in all the years of visual survey data included in this analysis (Fig 5c). These sightings suggest Gervais’ presence much further north than indicated by our acoustic data, though northerly sightings were located much farther offshore than our recording devices, which may explain why there was no meaningful acoustic presence of Gervais’ north of Hatteras. Alternatively, some of these putative Gervais’ sightings may be mislabeled due to the difficulty of visually discriminating between mesoplodont beaked whales at sea, and potential misidentification of True’s beaked whales.

### True’s beaked whale (*Mesoplodon mirus*)

#### Description

**Fig 6:**
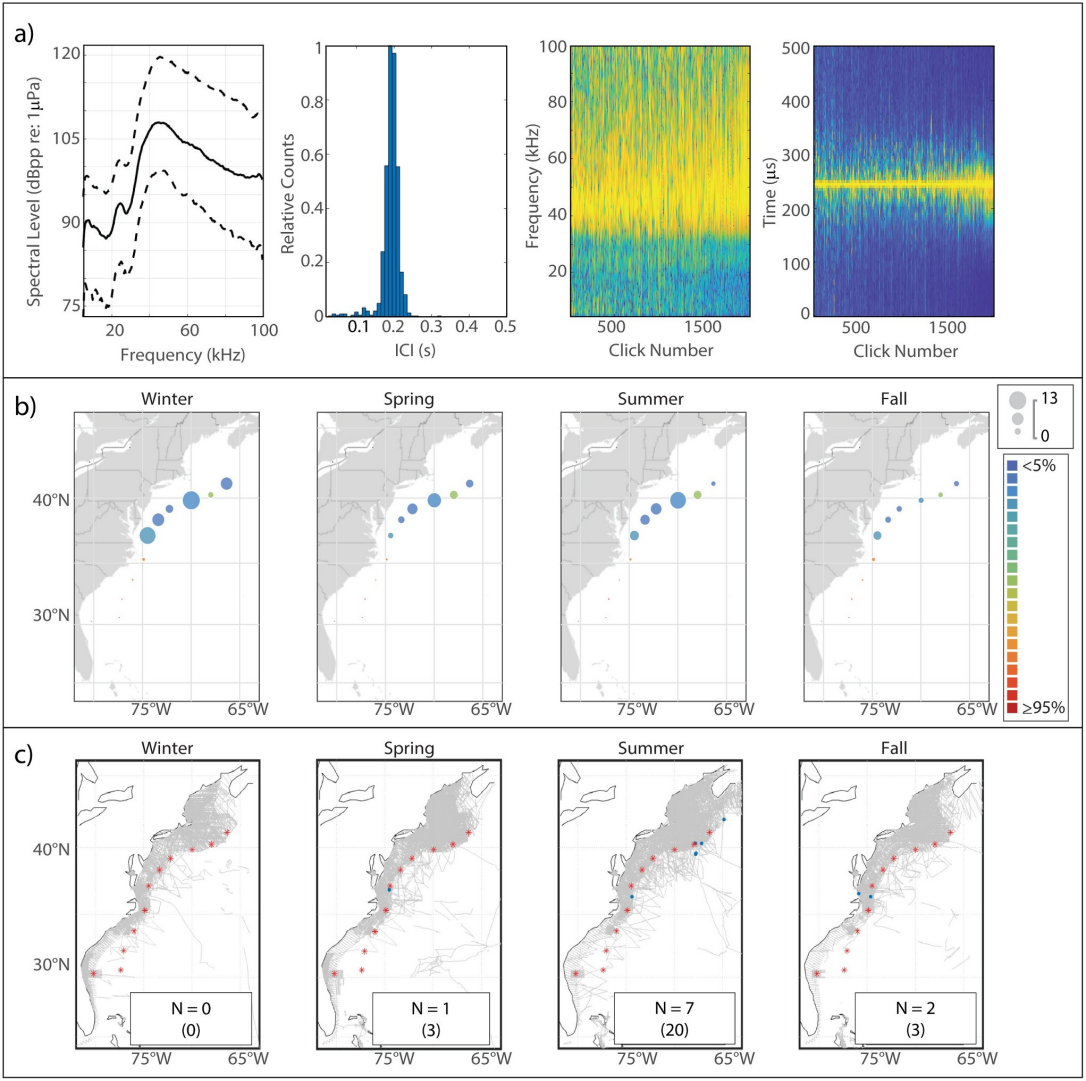
Results for True’s beaked whale showing click type (a), acoustic presence (b), and historical sightings (c). Subplots as in Fig 2.

True’s beaked whales produce clicks with a spectral shape similar to those of Gervais’ beaked whales, with a sharp onset of energy around 30 kHz [34]; the True’s clicks in our analysis reached peak amplitude at a median frequency of 48 kHz, with a much lower amplitude peak present at 24.5 kHz (Fig 6a). True’s beaked whale clicks can be distinguished from Gervais’ by a shorter median modal ICI value of 0.185 s.

#### Spatiotemporal distribution

True’s beaked whale clicks were detected at very low levels at all monitoring sites north of Hatteras (Fig 6b). A clear seasonal pattern was visible, with increased presence in both the summer and the winter compared to the fall and the spring, and lowest overall presence in the fall. Highest acoustic presence was seen at NC in all seasons but the fall. Sightings of True’s beaked whales were exceedingly rare, with just 10 records in all the years of visual survey data included in this analysis (Fig 6c), all of which occurred north of Hatteras.

### Cuvier’s beaked whale (*Ziphius cavirostris*)

#### Description

**Fig 7:**
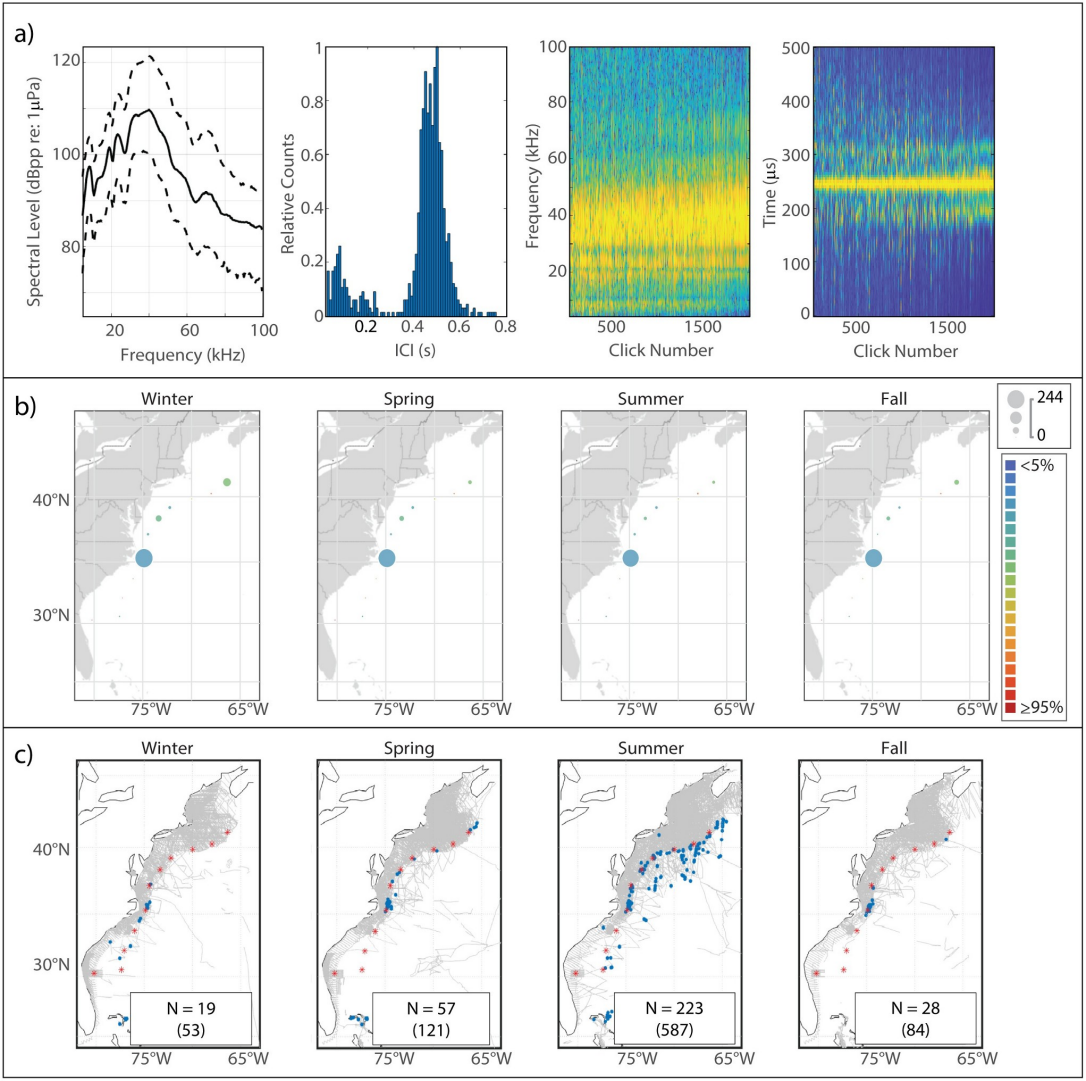
Results for Cuvier’s beaked whale showing click type (a), acoustic presence (b), and historical sightings (c). Subplots as in Fig 2.

The click type attributable to Cuvier’s beaked whale is distinctively multi-peaked [5, 49] (Fig 7a). The median peak frequency of Cuvier’s clicks in our analysis (38 kHz) doesn’t adequately describe the complex spectral shape, in which most of the click’s energy is focused in the main peak, but auxiliary peaks of successively decreasing amplitudes at ~23.5 kHz, ~19 kHz, and ~72 kHz were also consistently present. In our analysis this species exhibited a median modal ICI of 0.465 s.

#### Spatiotemporal distribution

The acoustic presence of Cuvier’s across our monitoring sites was focused at HAT, with low levels of presence north of this point and negligible presence at the southern sites (Fig 7b). A slight increase in presence at WC and HZ was visible in the winter. Sightings of Cuvier’s occurred mostly in the summer, with the majority of sightings along the shelf break and in deep offshore waters from Cape Hatteras north to Georges Bank (Fig 7c).

### *Kogia spp*.

#### Description

**Fig 8:**
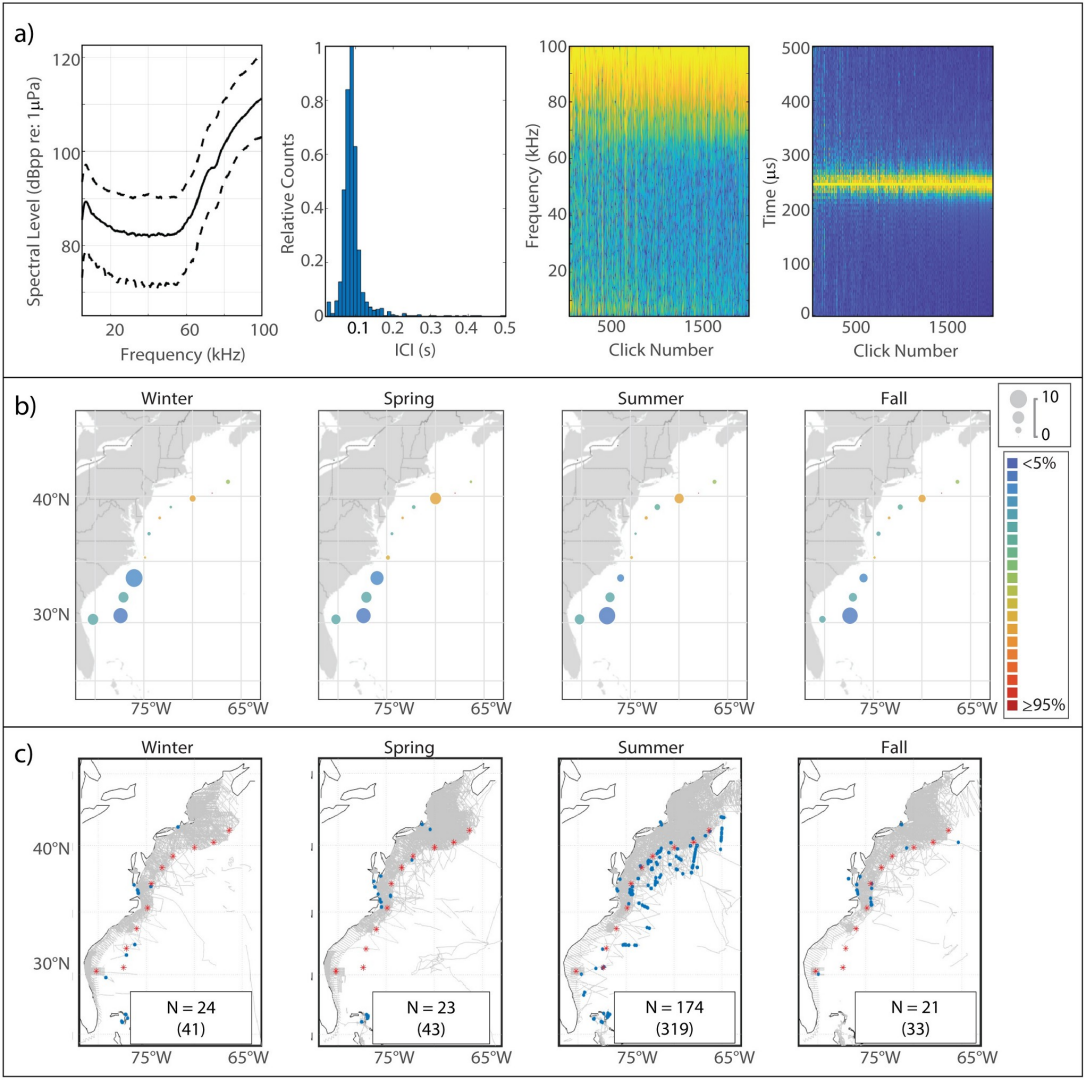
Results for *Kogia spp*. showing click type (a), acoustic presence (b), and historical sightings (c). Subplots as in Fig 2.

This high frequency click type is generated by both species in the genus *Kogia* [4, 50, 51]. The frequency content of these clicks was only partially captured by our sampling frequency of 200 kHz, and resultant Nyquist frequency of 100 kHz, but the energy distribution exclusively >60 kHz makes even a partial spectrum of this click type easily identifiable (Fig 8a). The median modal ICI value for *Kogia* clicks in our analysis was 0.085 s.

#### Spatiotemporal distribution

The overall acoustic presence of *Kogia spp*. at our monitoring sites was the lowest of all click types in our analysis and the distribution of this click type was strongly southerly, with highest presence at the four sites in the South Atlantic Bight (Fig 8b). A seasonal signal was visible at GS, with increased presence in the winter and spring compared to the summer and fall, but presence at the other southern sites was fairly consistent across seasons. Very low levels of true presence were coupled with high levels of error at the northern sites; the apparent increase in presence at NC was mostly due to a persistent high-frequency noise source occurring throughout the 2016–2017 deployment, which was misclassified as *Kogia spp*. Sightings of *Kogia spp*. occurred mostly in the summer, with the majority of sightings occurring along the shelf break and in deep offshore waters from Cape Hatteras north to Georges Bank (Fig 8c).

### Sperm whale (*Physeter macrocephalus*)

#### Description

**Fig 9:**
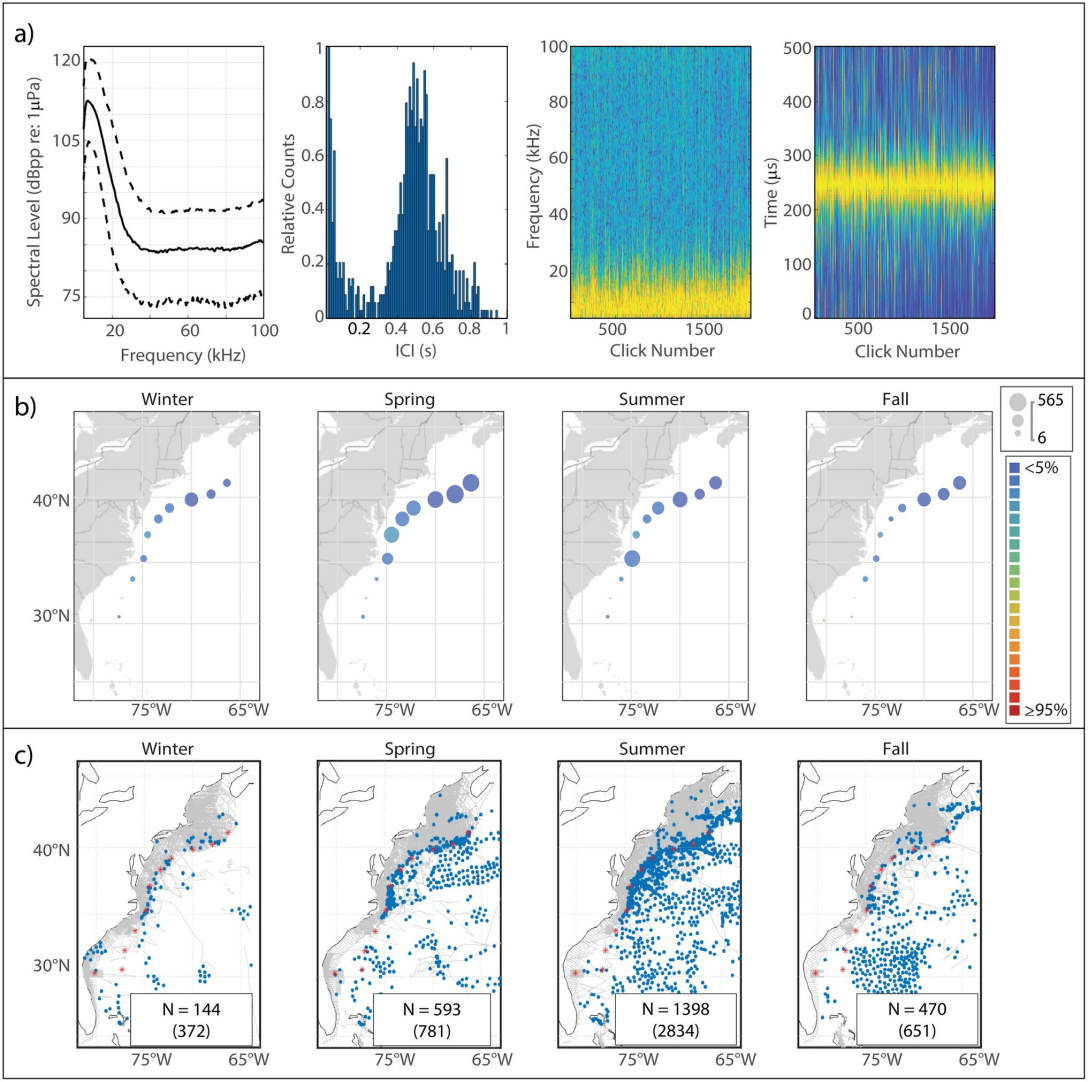
Results for sperm whale showing click type (a), acoustic presence (b), and historical sightings (c). Subplots as in Fig 2.

Sperm whale clicks are characterized by their low frequency content [3] (Fig 9a). The median peak frequency for sperm whale clicks in our study was 8.5 kHz, but it should be noted that this may have been skewed by our choice of a bandpass filter with passband from 5 kHz– 100 kHz, and the decision within the detector to exclude impulses with peak frequency <5 kHz. The median modal ICI value of 0.485 s was similar to what has been previously reported for female sperm whales [36, 37].

#### Spatiotemporal distribution

Sperm whales were the second most abundant click type in our analysis and were detected at all of our monitoring sites, with most presence detected from HAT northward (Fig 9b). An increase in presence was apparent across the northern sites in the spring, and lowest overall presence was seen in the winter. This pattern of acoustic presence was a good match for the distribution of historical sightings of sperm whales, which occurred primarily along the shelf break and in deep offshore waters, and were more numerous north of Cape Hatteras in the spring and summer months (Fig 9c).

### UD36—Risso’s dolphin (*Grampus griseus*)

#### Description

**Fig 10:**
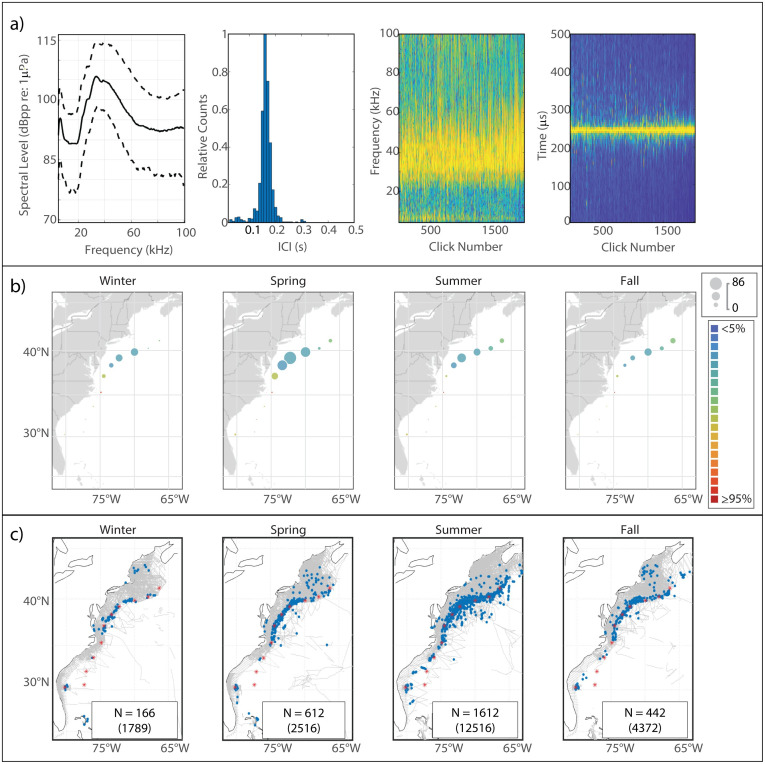
Results for UD36 showing click type (a), acoustic presence (b), and historical sightings of probable species match, Risso’s dolphin (c). Subplots as in Fig 2.

This click type was established based on clusters from several of the northern HARP sites which exhibited spectra with a main peak at 36 kHz characterized by a small trough, a lower amplitude peak at 26 kHz, and a shoulder at 23 kHz (Fig 10a). The median modal ICI value was 0.155 s. The UD36 click type shared several features, such as the location of spectral peaks and the ICI, with the click type identified in this dataset which was attributable to Risso’s dolphin (Fig 2a). The key difference was that the lower-frequency peaks of UD36 were not as pronounced as those present in the Risso’s click type.

#### Spatiotemporal distribution

UD36 exhibited a distinctive northerly distribution with highest presence at the WC, BC, and NC monitoring sites (Fig 10b). There was a marked increase in presence during the spring months which seemed to carry slightly into summer, with much lower levels of presence in the fall and winter. The distribution and seasonal pattern were very similar to the distribution of historical sightings of Risso’s dolphin (Fig 10c). During manual review of the automated labels we observed that UD36 was mostly confused with the Risso’s click type, and to a much lesser extent with UD38. We also observed that UD36 predominantly occurred interspersed throughout encounters with the Risso’s click type; high-quality encounters solely with UD36 did occur, however. This may suggest that UD36 is an alternative Risso’s click type, or that it is generated by a species which is often, but not always, associated with Risso’s dolphins. Due to the similarities in spectral shape and click rate we believe UD36 is likely an alternative Risso’s click type. Multiple click types have previously been reported for a single odontocete species [7, 29, 31, 32]; use of different click types may be determined by behavioral state, or may be a function of angle of arrival at the receiver or of regional variation [7, 52, 53].

### UD26—Short-finned pilot whale (*Globicephala macrorhynchus*)

#### Description

**Fig 11:**
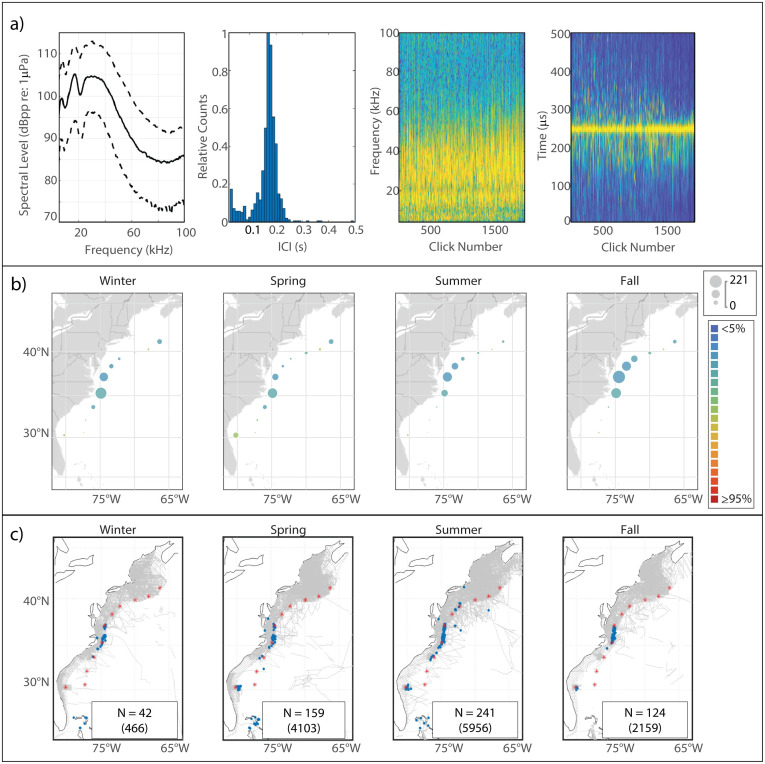
Results for UD26 showing click type (a), acoustic presence (b), and historical sightings of probable species match, short-finned pilot whales (c). Subplots as in Fig 2.

This click type had substantial low-frequency (<20 kHz) energy, with a double peaked structure characterized by a deep notch whose minimum fell between 20 kHz—23 kHz (Fig 11a). The narrow lower peak reached maximum amplitude typically around 19 kHz, while the broader upper peak extended from 25 kHz– 35 kHz. The median modal ICI value was 0.165 s. The low frequency content and relatively long ICI were consistent with a larger-bodied delphinid, such as the species in the subfamily *Globicephalinae*, commonly referred to as “blackfish”.

#### Spatiotemporal distribution

UD26 was predominantly found at and north of Cape Hatteras, between the HAT and BC monitoring sites (Fig 11b). This type exhibited a seasonal shift in presence, with higher presence at NFC and WC beginning in the summer and peaking in the fall, which gave way to higher presence at HAT beginning in the fall, peaking in the winter, and carrying into the spring. The regional distribution and seasonal presence of UD26 were a good match for the historical distribution of short-finned pilot whale sightings in this region (Fig 11c). The low overall acoustic presence of this click type was also in line with the relatively small number of sightings of short-finned pilot whales across all years of visual survey data. The only anomalous feature in this match was the presence of UD26 detections with relatively low error rates at HZ, as short-finned pilot whales are not thought to be present this far north. The detections labeled as UD26 at HZ showed a slight upwards shift in frequency content relative to the UD26 detections from the US mid-Atlantic region, but otherwise had a similar spectral shape and modal ICI. It may be that this northern variant of UD26 is in fact distinct from the UD26 encountered further south, and should be studied separately.

#### Supporting observations

**Fig 12:**
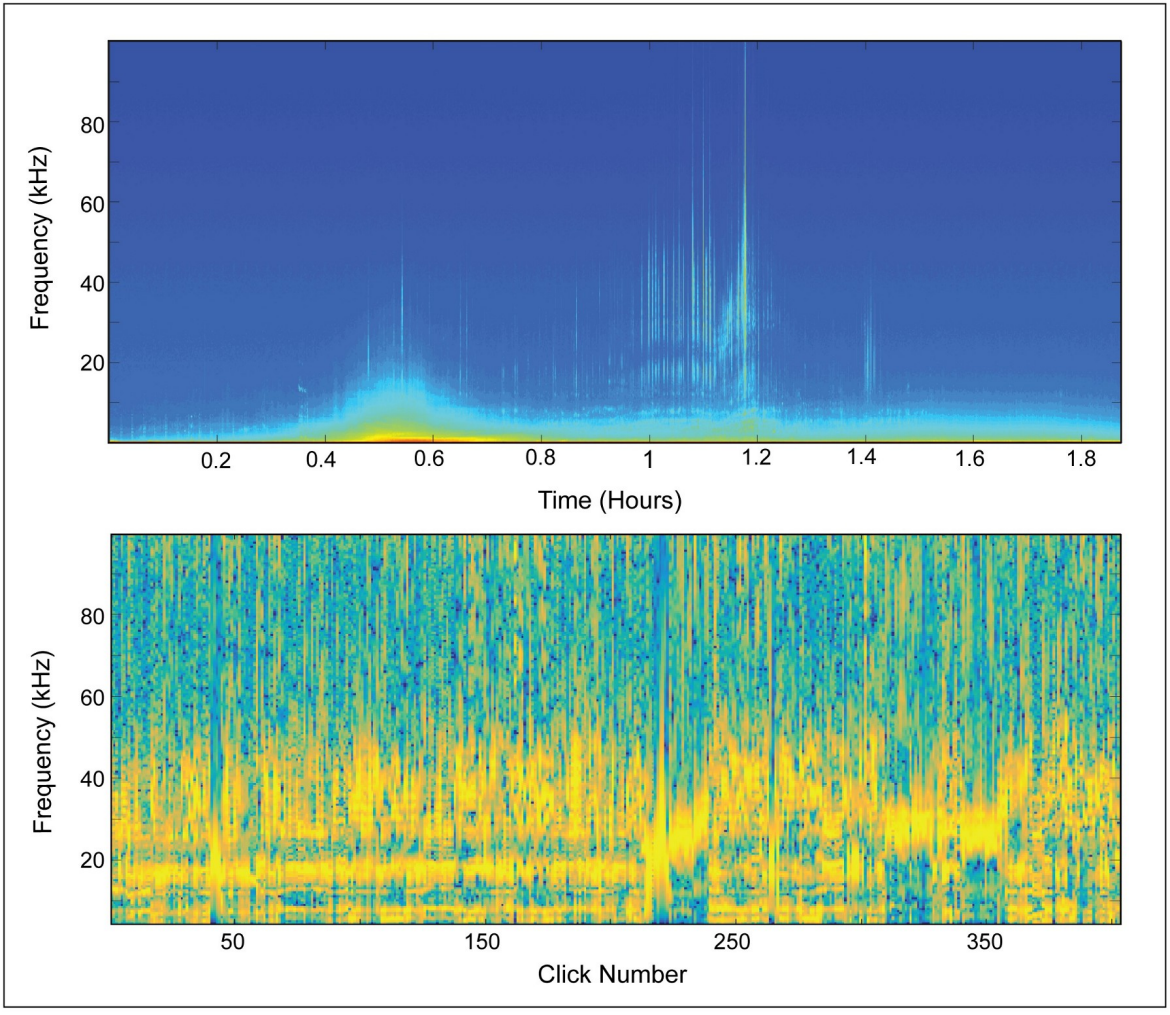
Visually confirmed short-finned pilot whale bout. Top panel: long-term spectrogram showing an acoustic encounter with visually-identified short-finned pilot whales at the JAX acoustic monitoring site. Bottom panel: concatenated spectra of clicks detected between 00:59 and 01:07. Most of these clicks exhibit spectral features consistent with UD26, though some natural variability is visible. In both plots the magnitude of the frequency is represented by color such that warmer colors show greater magnitude.

We identified three short-finned pilot whale sightings in close proximity to an acoustic device and looked at the concurrent acoustic data to identify any acoustic encounters which might be attributed to the sighted species. Acoustic encounters associated with two of the three sightings exhibited features consistent with those of UD26; one of these encounters, from JAX, is shown in Fig 12. The third encounter, which was very low amplitude, did not exhibit the characteristics of UD26. There was also a fourth sighting, just 0.63 km from NFC in October of 2017, which was associated with a high-amplitude encounter which strongly exhibited the characteristics of UD26; however, this visual sighting was only identified to the genus level. The scarcity of long-finned pilot whale sightings near NFC in the fall (S2 Fig) suggests that the species sighted during this fourth encounter was most likely short-finned pilot whale. Examination of acoustic encounters labeled as UD26 also revealed the consistent presence of low-frequency whistles (<10 kHz) and buzz-type calls previously reported for pilot whales [54, 55]. Additionally, a similar click type has been reported for short-finned pilot whales from Hawaii [33], the Gulf of Mexico [18], the western North Atlantic [56], and the eastern North Atlantic [45].

Manual review of a subset of the automated labels revealed that at sites where this type was more abundant, most of the classification error could be attributed to misclassification as UD19, which we believe may be another *Globicephalinae spp*. type. Such confusion occurred when the lower-frequency peak of UD26 was much higher-amplitude than the higher-frequency peak, resulting in a spectral shape quite similar to that of UD19. Our observations of clicks with a spectrum intermediate between UD26 and UD19 may indicate that short-finned pilot whales produce a variety of clicks describing a continuum between these two types. Alternatively, the frequent co-occurrence of these two types may tell us that the short-finned pilot whales producing UD26 sometimes co-occur with other *Globicephalinae* species producing UD19.

### UD28—Short-beaked common dolphin (*Delphinus delphis*)

#### Description

**Fig 13:**
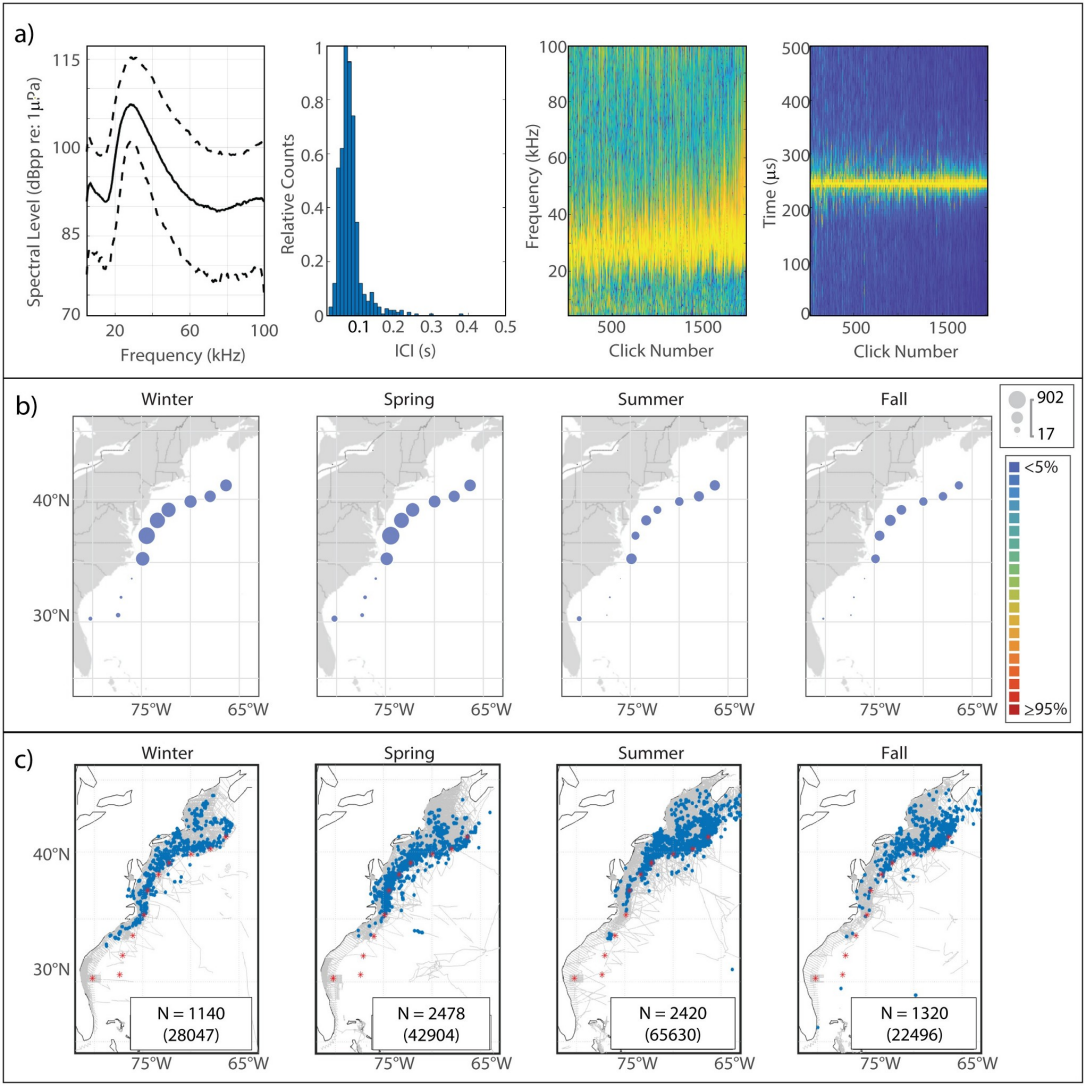
Results for UD28 showing click type (a), acoustic presence (b), and historical sightings of probable species match, short-beaked common dolphin (c). Subplots as in Fig 2.

This click type had a simple spectral structure with a single peak around 28 kHz (Fig 13a) and a short median modal ICI value of 0.075 s. Based on this generic shape, and the ubiquity of this click type across sites, UD28 seemed likely attributable to bottlenose or short-beaked common dolphins, both of which were common in the study area.

#### Spatiotemporal distribution

This click type was the most abundant type detected in our analysis and exhibited the lowest classification error across sites of all the novel types (Fig 13b). UD28’s distribution predominantly north of Cape Hatteras, with increased presence between HAT and BC in the winter and spring months, was highly similar to the historical distribution of short-beaked common dolphin sightings in this region (Fig 13c). Similarly generic click spectra have also been previously reported for bottlenose dolphins [30, 52], but the abundance of UD28 at the northern sites in the winter does not mirror the distribution of bottlenose dolphin sightings in this region (S5 Fig).

#### Supporting observations

A click type similar to UD28 has been previously reported for short-beaked common dolphin clicks in the Pacific [6]. Additionally, the long duration and dense clicking activity typical of UD28 bouts in this study region suggests large group sizes. According to the sighting data we compiled, this is more in keeping with what has been observed for short-beaked common dolphins (mean group size: 30.7 individuals, 10^th^ & 90^th^ percentiles: [1, 60]; from 5183 sightings with group size data recorded) than for bottlenose dolphins (mean group size: 7.6 individuals, 10^th^ & 90^th^ percentiles: [1,18]; from 26,086 sightings with group size data recorded).

### UD19—*Globicephalinae spp*.

#### Description

**Fig 14:**
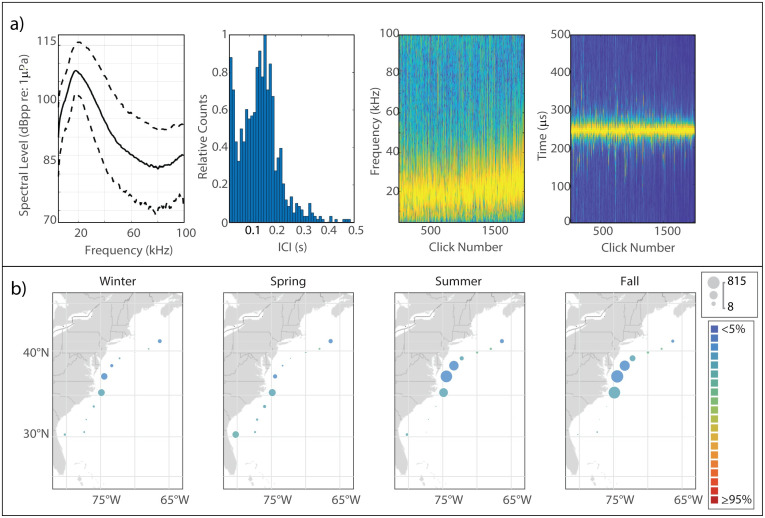
Results for UD19 showing click type (a), and acoustic presence (b). Subplots as in Fig 2a and 2b.

UD19 had a simple spectral shape similar to UD28, but with the peak centered at a lower frequency of 19 kHz (Fig 14a). The modal ICI value for this click type was 0.135 s. Similar to UD26, the low frequency and slow click rate may be indicative of a large-bodied species within the subfamily *Globicephalinae*.

#### Spatiotemporal distribution

This click type was the third most abundant in our analysis, after UD28 and sperm whales, and was present at all sites at least part of the year (Fig 14b). UD19 showed a pronounced seasonal pattern with highest presence at NFC and WC in the summer and fall, and much lower levels of presence everywhere in the winter and spring. However, the distribution and seasonal pattern of this click type were not good matches for the distribution of sighting data for any single dolphin species found in this region, *Globicephalinae spp*. or otherwise. There were some similarities in the seasonal distribution of UD19 to that of UD26, suggesting at first glance a match for short-finned pilot whales, but this may to some extent have been due to confusion between UD26 and UD19. As described above, clicks spanning a continuum between these two spectral shapes were often observed during bouts with both UD26 and UD19, resulting in confusion consistent with what was seen during classifier testing (S1 Table). There was some ambiguity as to the best choice of “true” class for clicks with a pronounced main peak at 19 kHz in addition to a much lower amplitude auxiliary peak between 25 kHz—30 kHz. Even after accounting for the incidence of false positive UD19 detections, the abundance of UD19 was incongruous with the low number of short-finned pilot whale sightings in this area (Fig 11c). The more numerous *Globicephalinae* species in the study region was the long-finned pilot whale, but the distribution of UD19 did not reproduce the distribution or seasonal patterns visible in long-finned pilot whale sightings (S2 Fig). One possible explanation for this ambiguity is that UD19 does not represent a single species but may in fact be attributable to several *Globicephalinae* species which produce similar clicks and which have been inadvertently grouped into a single class in this analysis. In addition to long-finned pilot whales, short-finned pilot whales, orcas, false killer whales, pygmy killer whales, and melon-headed whales are also known to be present in the study area. The pooling of species with markedly different spatial distribution patterns may have resulted in a generalized distribution of this click type which obscures the distinct patterns of each species included.

#### Supporting observations

Similar to UD26, examination of encounters with UD19 revealed the consistent presence of low-frequency whistles (<10 kHz) and buzz-type calls typical of *Globicephalinae* species [54, 55].

### UD47—Distinctive type without a clear spatiotemporal match

#### Description

**Fig 15:**
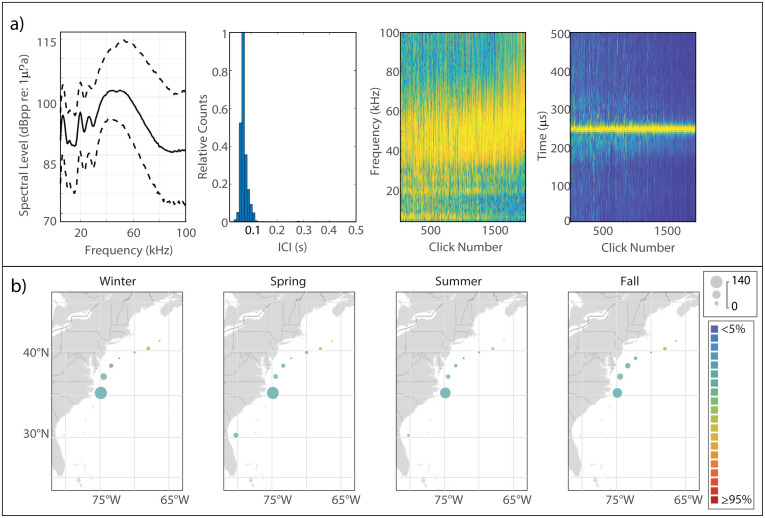
Results for UD47 showing click type (a), and acoustic presence (b). Subplots as in Fig 2a and 2b.

This click type was characterized by its distinctive spectral banding pattern, with well-defined low-amplitude peaks at 20 kHz and 28 kHz and a broad main peak between 40 kHz—55 kHz (Fig 15a). The modal ICI (0.065 s) was typical of smaller-bodied delphinids, of which there are several species in this region which have yet to be matched with a characteristic click type: bottlenose dolphins, Atlantic white-sided dolphin, white-beaked dolphin, Fraser’s dolphin, Atlantic spotted dolphin, pantropical spotted dolphin, spinner dolphin, Clymene dolphin, striped dolphin, rough-toothed dolphin.

#### Spatiotemporal distribution

UD47 exhibited negligible presence at the shelf break sites south of HAT, low levels of presence at JAX in the spring and summer, highest presence at HAT, and low levels of presence at the northern sites (Fig 15b). Very little seasonal pattern was apparent, though there was a slight increase in presence at HAT in the winter and spring months. This distribution was not a good match for the distribution of historical sighting data for any dolphin species in this region.

### UD38—Distinctive type without a clear spatiotemporal match

#### Description

**Fig 16:**
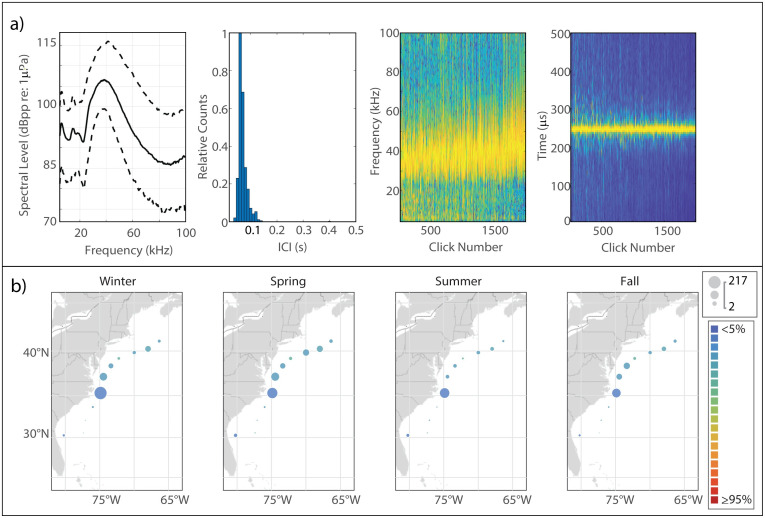
Results for UD38 showing click type (a), and acoustic presence (b). Subplots as in Fig 2a and 2b.

UD38 had a relatively narrow main peak with most energy between 38 kHz– 45 kHz, and two lower-amplitude auxiliary peaks at 16 kHz and 19 kHz; both lower-frequency peaks were not always apparent (Fig 16a). The modal ICI value was 0.065 s (Table 2); as with UD47, this may suggest a small-bodied delphinid.

#### Spatiotemporal distribution

UD38 exhibited a predominantly northerly distribution with highest presence always at HAT (Fig 16b). Presence at HAT peaked in the winter, while a slight increase in presence at the northern sites could be seen in the spring. There were low levels of acoustic presence of UD38 at the southern sites with variable error rates. Like UD47, there was no clear species match based on the distribution and seasonal pattern for this click type.

## Discussion

Our two-pronged approach leveraged big acoustic data and many decades of visual survey efforts to yield new inferences about odontocete acoustic identity, and was made possible by the combined power of automated algorithms and expert analyst review. Identification of six novel delphinid click types, and attribution of four of the six to a particular species/genus, substantially expands our ability to identify delphinid species presence in passive acoustic data from this region, and thereby pursue ecological studies. This approach can be applied for signal type discovery and identification in any region where large passive acoustic and visual survey data sets have been collected, and will enable improved utilization of large marine passive acoustic data sets. The catalog of impulsive signal types presented here in the form of our neural network training classes is, to the best of our knowledge, the first of its kind for this area and represents a comprehensive overview of the dominant odontocete species and impulsive noise sources commonly found at deep water acoustic monitoring sites spanning the region.

Odontocetes produce directional clicks with greater amplitudes on-axis (forward of the rostrum) and lower amplitudes off-axis (lateral from the rostrum) [1]. Since our detector output did not discriminate between on-axis and off-axis clicks, the click types presented in this analysis may represent both on-axis and off-axis arrivals at our sensors. Previous works have suggested that most delphinid clicks arriving at a seafloor sensor are off-axis [42], while those of beaked whales are likely on-axis when the animals are more than a few hundred meters from the sensor [38]. Clicks which arrive at a sensor from an off-axis path are typically distorted relative to their on-axis counterparts, with complex waveforms, amplitude and peak frequency decreasing as a function of off-axis angle, and spectral notches often being introduced [7, 52, 57–59]. Angle of off-axis is also an important consideration, as click which are only slightly off-axis may appear very similar to on-axis clicks. In a passive acoustic monitoring paradigm it is reasonable to assume that a large proportion of clicks arriving on a sensor are off-axis, but this did not appear to be a reason, in and of itself, to discard these clicks from analysis. Off-axis click have generally not been as well-studied as on-axis clicks but their distortions may carry a signature of the acoustic anatomy of the generating species, and therefore there may be species-specific features of off-axis clicks which make them equally well suited to species classifications as on-axis clicks [6]. If some of the click types presented here represent off-axis arrivals, this may explain why the species to click type correspondence is not always one to one, both in this work and in previous works [29, 31, 32]. A better understanding of the relationship between on-axis and off-axis click features as they are received by a seafloor sensor would be valuable for improved interpretation of large passive acoustic data sets. This could perhaps be obtained through studies combining body-mounted orientation-recording tags and seafloor acoustic sensors.

The distribution patterns exhibited by the known click types we identified represent two distinct cases: in the case of Risso’s dolphins and sperm whales, the acoustic presence mirrors the distribution and seasonal patterns of sightings along the shelf break (Figs 2b, 2c, 9b and 9c), whereas in the case of the beaked whales and *Kogia spp*., the acoustic data reveals presence patterns which are not represented in the sighting data (Figs 3–8b and 8c). The former case is an encouraging proof-of-concept for our approach of matching acoustic presence patterns to the distribution of historical sighting data in order to attribute novel click types to species. For species which are readily available for both visual detection and acoustic detection, the two approaches should generate comparable presence maps, and we see this in the result for both Risso’s dolphins and sperm whales. In the latter case we see a mismatch between the presence patterns captured by the two methodologies, which suggests that one of these approaches is not well-suited to detecting the species of interest. Indeed, beaked whales and *Kogia spp*. are known to be cryptic species which exhibit inconspicuous surface behaviors and undertake prolonged deep dives, complicating the task of inferring species presence patterns from ship-based and aerial sighting data. Autonomous passive acoustic data collection captures animals throughout the water column and is thought to have no effect on animal presence or behavior, so the acoustic presence recorded via this methodology may be a better indicator of the true spatiotemporal presence patterns of these elusive species. This is likely the case for Sowerby’s, Blainville’s, Gervais’, and True’s beaked whales, as the acoustic presence maps suggest that they are present at more sites and throughout more seasons than is shown by the historical sighting data (Figs 3–6b and 6c). In each case there are, however, some offshore sightings which represent animals which would not have been available to be captured in the acoustic data due to their distance from the acoustic monitoring sites, demonstrating the limitations of point sampling compared to data collection along far-reaching track lines. For Cuvier’s beaked whales and *Kogia spp*. these missed presence points are fairly numerous in the summer months (Figs 7c and 8c). However, the fact that there is no corresponding increase in acoustic presence of either species in the northern region during the summer may suggest that the increase in sightings is due to disproportionate summer survey effort, rather than a true increase in presence. The fullest understanding of the distribution and seasonal presence patterns for these two species could likely be attained by combining passive acoustic and visual survey data.

Matching acoustic presence maps to historical sighting maps requires consideration of the differences between these two approaches to observing species presence. UD36, the novel click type assigned to Risso’s dolphin, exhibited a clear increase in acoustic presence in the spring, which mirrored the increased density in Risso’s sightings tightly clustered along the shelf break north of Hatteras in the spring compared to the winter and fall (Fig 10b and 10c). However, in absolute terms there were more Risso’s individuals sighted in the summer. These summer sightings were somewhat more widely distributed on the shelf and offshore, meaning many individuals were too far from our devices to be acoustically detected, which may explain why this increase was not as clearly reflected in the summertime acoustic presence of UD36 at our monitoring sites. Increased summer Risso’s sightings may also be a function of increased sighting effort, as opposed to increased species presence, while acoustic monitoring effort was uniform throughout seasons. Similar to UD36, the acoustic presence of UD28, assigned here to short-beaked common dolphins, exhibited increased presence in the winter and spring, while short-beaked common dolphin sightings peak in the summer (Fig 13b and 13c). This may be due to the apparent shift in distribution of short-beaked common dolphins, from the outer shelf and shelf break during the winter and spring northward onto Georges Bank and inshore during the summer and fall. Many of the animals sighted in the summer and fall were not available to be captured on our acoustic devices for this reason. The distribution of short-finned pilot whale sightings (Fig 11c) may underestimate their true presence due to missing data points, as many *Globicephala* sightings are identified only to the genus level (2701 *Globicephala spp*. records in our analysis, compared to 566 *G*. *macrorhynchus* and 1361 *G*. *melas* records). In areas where the ranges of the two pilot whale species overlap, high probabilities of sighting either species may lead to lower confidence in species level identifications, and more sightings reported simply as *Globicephala spp*. (S2 Fig). The predominance of UD26 clicks during multiple encounters with visually-confirmed short-finned pilot whales complements the possibly-incomplete sighting data to support this species assignment.

UD19 exhibited a very strong summer and fall presence at the mid-Atlantic sites, but it is unclear from this analysis where these individuals, which we believe may represent more than one species, spend the winter and spring months (Fig 14b). It may be that the individuals accounting for the high levels of acoustic presence at HAT, NFC, and WC in the summer and fall are a different species than the individuals accounting for the lower levels of presence across all sites in the winter and spring. Further study of the variability within this type may reveal subtypes with varying seasonal presence which could be linked to the presence of particular species. UD47 and UD38 may be attributable to species whose distribution and seasonal presence patterns are not well-elucidated by historical sighting data, and identification of these click types to species may necessitate additional data types.

The oceanography in this region is dominated by the influence of the Gulf Stream, a high-volume current which transports warm, high-salinity equatorial water along the shelf break of the southeastern U.S. until its separation point at Cape Hatteras, where it turns eastward towards northern Europe. The Gulf Stream front is a steep gradient in temperature and salinity which delineates a boundary between two very different habitats: warm oligotrophic waters of the Gulf Stream to the south and east, and cold, lower-salinity, productive sub-polar waters to the north and west. It is not surprising, therefore, that the distributions of many species in this region reflect the presence of this boundary. Most of our unidentified click types exhibited little to no presence at the three monitoring sites situated directly in the path of the Gulf Stream: GS, BP and BS. As can be seen by the presence of Blainville’s and Gervais’ beaked whales and *Kogia spp*. acoustic encounters at these sites (Figs 4c, 5c and 8c), this was not likely a result of poor acoustic propagation conditions or low detectability at these sites, but rather a clear species preference regarding the conditions of the Gulf Stream waters. The distinct regional and temporal patterns exhibited by the distributions of each of the unidentified click types may provide us with insights into the ecology of the species to which they are attributed.

### Clustering and click type identification

Even with the help of the automated signal discovery pipeline, the process of identifying recurring signal types across so many sites and years of data was not a trivial matter. A consideration when using an automated clustering approach is the tradeoff between cluster separation and the proportion of nodes (5-minute bin average spectra, in this analysis) which are isolated by user-defined thresholds. By tuning the parameters of the clustering process we are able to impose arbitrary requirements of cluster size and self-similarity, yielding types which describe highly consistent and commonly present signals. This comes at the expense of types which exhibit greater natural variability or are present in lower numbers. The first case may be acceptable for our purposes, which rely upon consistently present spectral and temporal features to discriminate between species. The loss of rare types, however, undermines our efforts to identify characteristic click types for these species. When comparing clusters across sites, the requirement for apparent click types to exhibit presence at multiple sites and across the years of recording effort (in order to avoid establishing types based on site-specific noise sources or atypical species presence phenomena) likely further excluded species with low or intermittent presence in this region, even if they did have highly distinctive and recognizable clicks which formed robust clusters. This is an issue worth exploring further, as rare species are often the most data deficient, and methods of collecting information on their presence and distribution are sorely lacking. Further study of the clusters excluded from our training set may reveal types which correlate well with the presence of rare species. Future work should explore approaches to identifying rare click types in these large data sets and differentiating them from random noise events.

After acceptably self-similar clusters had been formed, the issue of signal variability was still highly pertinent when comparing clusters and making decisions about which should be deemed examples of a single type, and which warranted separate consideration. As with all manual signal identification, this step involved subjective judgement calls guided by knowledge of previously documented signal types and the characteristics of our monitoring sites. Due to the lack of supporting data justifying subdivisions of similar clusters, an approach favoring simplicity over hyper-fragmentation of types was chosen. This may have resulted in signal categories which obscured some species-level differences, such as may be the case for UD19. We did not, however, choose to merge UD36 with the Risso’s click type despite their apparent similarity for two reasons: 1) while these types often co-occurred in our data, high quality encounters with solely UD36 were also present, and, 2) UD36 has not been observed in click clusters generated from HARP data from the Gulf of Mexico [18] or southern California (analyses underway), where Risso’s are regularly acoustically detected. This suggested to us that UD36 might actually be generated by a species other than Risso’s dolphin, or might be a regionally-specific click type indicative of stock delineations [53]. For these reasons we initially chose to analyze UD36 independent of the previously established Risso’s click type. An alternative to this manual approach to establishing click types would be to carry out a third clustering step, comparing clusters across deployments and sites in an automated fashion as opposed to manually. It should be noted that at sites where multiple odontocete species frequently co-occur (most notably HAT and NFC), the clusters themselves may not always have been single-species. In cases of the co-occurrence of species with similar click types, multiple spectra may have been separated within each 5-minute bin by the first step of clustering, but then any combination of those bin-level spectra may have been included in a final cluster with characteristics spanning two or more highly similar species. Such an occurrence would mean that the different species were not effectively available to be separated during manual review of the clusters from that site (and neither would they be separated by a third pass of clustering).

### Classification error

During the classification step, the neural network was required to distinguish both highly divergent signals and quite similar signals, likely resulting in lower success in discriminating between similar signals. This could muddy the waters when looking at the geographic distribution of each click type. An iterative approach to labeling, where broad classes are first separated (i.e. sperm whale, beaked whales, dolphins) and then individual classifiers tuned to the more nuanced distinctions within each class are run in a second step, might show improved discriminatory ability. We did observe, however, that our classifier was resilient to small shifts in frequency content when the overall spectral shape was conserved, as seen in the classification of UD26 at mid-Atlantic versus northern sites. This is a useful quality when looking to discriminate to the species level, as regional differences in frequency content for a given species have been previously described [53], and these differences may be accommodated by the kind of classifier used here.

We found that classification error, quantified by the false positive rate for the novel click types, varied greatly between click types, and also between sites within some of the types. One of the challenges with multi-class classification is that the probability of successful classification is inversely proportional to the number of classes. For a classifier choosing between 20 classes, the probability of random success is just 5%; training data improves those odds substantially, but the model is still challenged by discriminating between so many classes, some of which are quite similar. The error rates reported here are typically much lower than would be expected from random guessing, except when presence of a click type at a given site is very low. Manual review of a subset of the labels, and observation of the presence of many intermediate and noisy clicks, drove home the impracticality of attempting manual labeling from scratch. Especially at sites where many species are present and acoustic bouts overlap, such as HAT and NFC, distinguishing intra-type variability from inter-type variability can perplex even highly specialized analysts, and requires a prodigious time investment. These are important factors to keep in mind when evaluating classifier accuracy and considering the tradeoff between accuracy and time required to generate labeled time series. The approach to signal classification used here is fast, objective, and repeatable, and there are many options available for continuing to improve the classifier, such as multi-step classification and model ensembles.

A recurring feature across many of our click types was higher levels of classifier error at sites with low levels of presence for that type. This is to be expected when there is a mismatch between the probability distribution of classes learned by the neural network and the probability distribution of species present in the data. This phenomenon, known as dataset shift, has recently gained attention in the literature on machine learning applications in ecological studies, along with some proposed solutions [60–63]. The insights gained here regarding the presence of different click types across our sites could be applied in future to create a training set which more accurately reflects the true probability distribution of each type.

We sought to minimize our classifier error by enforcing increasingly strict minimum peak-to-peak receive level and number-clicks-per-bin thresholds in order to weed out low-quality clicks. In the end, we found that this approach did not have much impact on the patterns in distribution and seasonal presence visible in the acoustic presence maps, though it did dramatically reduce the number of clicks retained for analysis. In light of this we decided to use fairly relaxed thresholds in order to retain more of the detected clicks in our analysis. Due to the number of classes evaluated here, the size of the acoustic data set, and the uncertainty involved in “true” class selection for intermediate clicks when quantifying confusion, we calculated a single FPR for each novel click type at each site, and used that single value to scale the acoustic presence in all seasons. Development of efficient and objective approaches to quantification of confusion in such large data sets when out-of-distribution signals are present could lead to more accurate time series adjustments, as well as an opportunity to improve future classifiers by identifying the particular features a classifier had difficulty separating.

## Conclusion

Our findings illustrate the complementary nature of marine passive acoustic and visual survey data, and provide a means of ascertaining species identity for novel acoustic signals within existing and forthcoming acoustic data sets based on spatiotemporal correlations. The workflow described here provides a highly objective, repeatable, and fast approach to signal discovery and classification for large acoustic data sets. Identification of two unidentified click types from this region as short-beaked common dolphins and short-finned pilot whales, as well as attribution of a second click type to Risso’s dolphin, expands our knowledge of species-specific click types and sets the stage for ecological studies of these species using passive acoustic data. Assignment of UD19 to the *Globicephalinae* subfamily is a first step in species identification, though more work remains to disentangle the ambiguity remaining around this click type. Species identities were not forthcoming for UD47 or UD38 in this analysis, but the recognition of these recurring signal types as likely delphinid click types will enable further study of their occurrence patterns, which may lead to future species identifications.

## Supporting information

S1 Table Neural network test performance on a balanced test set of 500 examples per class. Names for known-type classes are abbreviations of the species/genus names: *Md*: *Mesoplodon densirostris*; *Zc*: *Ziphius cavirostris*; *Me*: *Mesoplodon europaeus; GoM Me*: *Gulf of Mexico Mesoplodon europaeus; Kogia*: *Kogia spp*.; *Gg*: *Grampus griseus; Mb*: *Mesoplodon bidens; Pm*: *Physeter microcephalus; Mm*: *Mesoplodon mirus*.
(DOCX)

S1 Text Noise class descriptions. (DOCX)

S2 Text Sighting data citations. (DOCX)

S1 Fig Characteristics of noise classes; a) ships; b) mid-frequency sonar; c) high-frequency sonar; d) multi-frequency sonar; e) snapping shrimp. Columns are: median power spectrum (solid line) with 10^th^ and 90^th^ percentiles (dashed lines); distribution of modal IPI values from 1000 5-minute bins; concatenation of normalized impulsive signal spectra, sorted by received level; concatenation of normalized waveform envelopes, sorted by received level. For the concatenated spectra and waveform envelopes, the normalized magnitude of the frequency/pressure is represented by color such that warmer colors show greater magnitude.
(TIF)

S2 Fig Historical sighting maps of a) pygmy killer whales (*Feresa attenuata*); b) long-finned pilot whales (*Globicephala melas*); c) *Globicephalinae spp*.; d) Atlantic white-sided dolphins (*Lagenorhynchus acutus*); e) white-beaked dolphins (*Lagenorhynchus albirostris*). Sightings are plotted per season (blue dots), shown relative to acoustic monitoring sites (red stars) and track lines of surveys undertaken in each season (grey lines). Inset within each sighting map shows number of sightings; total number of individuals summed across all sightings for which group size data was available is given in parentheses.
(TIF)

S3 Fig Historical sighting maps of a) Fraser’s dolphin *(Lagenodelphis hosei*); b) killer whales (*Orcinus orca*); c) false killer whale (*Pseudorca crassidens*); d) melon-headed whale (*Peponocephala electra*); e) pantropical-spotted dolphins (*Stenella attenuata*). Sightings are plotted per season (blue dots), shown relative to acoustic monitoring sites (red stars) and track lines of surveys undertaken in each season (grey lines). Inset within each sighting map shows number of sightings; total number of individuals summed across all sightings for which group size data was available is given in parentheses.
(TIF)

S4 Fig Historical sighting maps of a) rough-toothed dolphins *(Steno bredanensis*); b) striped dolphins (*Stenella coeruleoalba*); c) Clymene dolphins (*Stenella clymene*); d) Atlantic-spotted dolphins (*Stenella frontalis*); e) spinner dolphins (*Stenella longirostris*). Sightings are plotted per season (blue dots), shown relative to acoustic monitoring sites (red stars) and track lines of surveys undertaken in each season (grey lines). Inset within each sighting map shows number of sightings; total number of individuals summed across all sightings for which group size data was available is given in parentheses.
(TIF)

S5 Fig Historical sighting map of bottlenose dolphins (Tursiops truncatus). Sightings are plotted per season (blue dots), shown relative to acoustic monitoring sites (red stars) and track lines of surveys undertaken in each season (grey lines). Inset within each sighting map shows number of sightings; total number of individuals summed across all sightings for which group size data was available is given in parentheses.
(TIF)
