# Supplementary material for: Identification of western North Atlantic odontocete echolocation click types using machine learning and spatiotemporal correlates
Source: PLoS One. 2022 Mar 24;17(3):e0264988. doi: 10.1371/journal.pone.0264988 (PMC8946748; doi:10.1371/journal.pone.0264988)
Supplement: S1 Table — Names for known-type classes are abbreviations of the species/genus names: Md: Mesoplodon densirostris; Zc: Ziphius cavirostris; Me: Mesoplodon europaeus; GoM Me: Gulf of Mexico Mesoplodon europaeus; Kogia: Kogia spp.; Gg: Grampus griseus; Mb: Mesoplodon bidens; Pm: Physeter microcephalus; Mm: Mesoplodon mirus. (DOCX) [file pone.0264988.s001.docx]

|  | ***Gg*** | ***Kogia*** | ***Mb*** | ***Md*** | ***Me*** | **GoM *Me*** | ***Mm*** | ***Pm*** | ***Zc*** | **UD36** | **UD26** | **UD28** | **UD19** | **UD47** | **UD38** | **Ships** | **High-Freq Sonar** | **Mid-Freq Sonar** | **Multi-Freq Sonar** | **Snap Shrimp** |
| --- | --- | --- | --- | --- | --- | --- | --- | --- | --- | --- | --- | --- | --- | --- | --- | --- | --- | --- | --- | --- |
| ***Gg*** | 91.8 | 0 | 0 | 0 | 0 | 0 | 0 | 0 | 0 | 8.2 | 0 | 0 | 0 | 0 | 0 | 0 | 0 | 0 | 0 | 0 |
| ***Kogia*** | 0 | 100 | 0 | 0 | 0 | 0 | 0 | 0 | 0 | 0 | 0 | 0 | 0 | 0 | 0 | 0 | 0 | 0 | 0 | 0 |
| ***Mb*** | 0 | 0 | 99.4 | 0 | 0 | 0 | 0.2 | 0 | 0 | 0 | 0 | 0 | 0 | 0.2 | 0 | 0 | 0.2 | 0 | 0 | 0 |
| ***Md*** | 0 | 0 | 0 | 100 | 0 | 0 | 0 | 0 | 0 | 0 | 0 | 0 | 0 | 0 | 0 | 0 | 0 | 0 | 0 | 0 |
| ***Me*** | 0 | 0 | 0 | 0 | 99.8 | 0 | 0.2 | 0 | 0 | 0 | 0 | 0 | 0 | 0 | 0 | 0 | 0 | 0 | 0 | 0 |
| **GoM *Me*** | 0 | 0 | 0 | 0 | 0 | 100 | 0 | 0 | 0 | 0 | 0 | 0 | 0 | 0 | 0 | 0 | 0 | 0 | 0 | 0 |
| ***Mm*** | 0 | 0 | 0 | 0 | 0 | 0 | 100 | 0 | 0 | 0 | 0 | 0 | 0 | 0 | 0 | 0 | 0 | 0 | 0 | 0 |
| ***Pm*** | 0 | 0 | 0 | 0 | 0 | 0 | 0 | 96 | 0 | 0 | 0 | 0 | 0 | 0 | 0 | 3.4 | 0.2 | 0.4 | 0 | 0 |
| ***Zc*** | 0 | 0 | 0 | 0 | 0 | 0 | 0 | 0 | 99.6 | 0 | 0 | 0 | 0 | 0 | 0.4 | 0 | 0 | 0 | 0 | 0 |
| **UD36** | 0.4 | 0 | 0 | 0 | 0 | 0 | 0 | 0 | 0 | 99.6 | 0 | 0 | 0 | 0 | 0 | 0 | 0 | 0 | 0 | 0 |
| **UD26** | 0 | 0 | 0 | 0 | 0 | 0 | 0 | 0 | 0 | 0 | 99.8 | 0 | 0.2 | 0 | 0 | 0 | 0 | 0 | 0 | 0 |
| **UD28** | 1 | 0 | 0 | 0 | 0 | 0 | 0 | 0 | 0 | 0.4 | 0.2 | 97.8 | 0.4 | 0 | 0.2 | 0 | 0 | 0 | 0 | 0 |
| **UD19** | 0 | 0 | 0 | 0 | 0 | 0 | 0 | 0 | 0 | 0 | 6.8 | 0.8 | 92.4 | 0 | 0 | 0 | 0 | 0 | 0 | 0 |
| **UD47** | 0 | 0 | 0 | 0 | 0 | 0 | 0 | 0 | 0 | 0 | 0 | 0 | 0 | 99.8 | 0.2 | 0 | 0 | 0 | 0 | 0 |
| **UD38** | 0 | 0 | 0 | 0 | 0 | 0 | 0 | 0 | 0.2 | 0 | 0 | 0 | 0 | 1 | 98.8 | 0 | 0 | 0 | 0 | 0 |
| **Ships** | 0 | 0 | 0 | 0 | 0 | 0 | 0 | 4.2 | 0 | 0 | 0 | 0 | 0 | 0 | 0 | 95.8 | 0 | 0 | 0 | 0 |
| **High-Freq Sonar** | 0 | 0 | 0 | 0 | 0 | 0 | 0 | 0 | 0 | 0 | 0 | 0 | 0 | 0 | 0 | 0 | 100 | 0 | 0 | 0 |
|  |  |  |  |  |  |  |  |  |  |  |  |  |  |  |  |  |  |  |  |  |
| **Mid-Freq Sonar** | 0 | 0 | 0 | 0 | 0 | 0 | 0 | 0 | 0 | 0 | 0 | 0 | 0 | 0 | 0 | 0.4 | 0 | 99.6 | 0 | 0 |
| **Multi-Freq Sonar** | 0 | 0 | 0 | 0 | 0 | 0 | 0 | 0 | 0 | 0 | 0 | 0 | 0 | 0 | 0 | 0 | 0 | 0 | 100 | 0 |
| **Snap Shrimp** | 0 | 0 | 0 | 0 | 0 | 0 | 0 | 0 | 0 | 0 | 0 | 0 | 0 | 0 | 0 | 0 | 0 | 0 | 0 | 100 |
