# Supplementary material for: Identification of western North Atlantic odontocete echolocation click types using machine learning and spatiotemporal correlates
Source: PLoS One. 2022 Mar 24;17(3):e0264988. doi: 10.1371/journal.pone.0264988 (PMC8946748; doi:10.1371/journal.pone.0264988)
Supplement: S1 Text — (DOCX) [file pone.0264988.s002.docx]

***Noise Class Descriptions***

**Ships** - Ship noise, produced through propeller cavitation and, to a lesser extent, mechanical noise from the operation of components such as engines, generators, fans, power plants, etc., exhibits a power spectrum with most energy in the 10-100Hz range, though energy may extend as high as 10kHz [1,2] (Fig. S1a). Since cavitation bubbles are not produced at a regular rate, the inter-impulse-interval distribution for boat noise is right-skewed.

**Mid-Frequency Sonar** - This class accounts for the presence of mid-frequency (1 kHz – 10 kHz) sonar at several of our southern sites (Fig. S1b). The inter-pulse-interval histogram for this class is likely multimodal due to the use of variable ping rates across different instances of the use of this sonar.

**High-Frequency Sonar** - This class was created as a catchall for a few types of high-frequency (>10 kHz) sonar observed in our data; the training examples included signals with peak frequencies at 14 kHz, 50 kHz, and 69 kHz (Fig. S1c). The inter-pulse-interval histogram for this class is likely multimodal due to the use of variable ping rates across different instances of the use of this sonar.

**Multi-Frequency Sonar** - This class accounts for the presence of what may be multi-beam sonar at some of our types; in the training examples for this class, multiple energy peaks are present simultaneously (Fig. S1d).

**Snapping Shrimp** - Snapping shrimp produce broadband impulses which look spectrally similar to echolocation clicks ([3], Fig. S1e). However, there is no coherent snapping rate, and so the inter-pulse-interval histogram exhibits a right-skewed distribution.

References:

1. Wenz GM. Acoustic Ambient Noise in the Ocean: Spectra and Sources. J Acoust Soc Am. 1962;34(12):1936–56.

2. Hildebrand JA. Anthropogenic and natural sources of ambient noise in the ocean. Mar Ecol Prog Ser. 2009;395:5–20.

3. Au WWL, Banks K. The acoustics of the snapping shrimp Synalpheus parneomeris in Kaneohe Bay . J Acoust Soc Am. 1998;103(1):41–7.
