# Supplementary material for: Identification of western North Atlantic odontocete echolocation click types using machine learning and spatiotemporal correlates
Source: PLoS One. 2022 Mar 24;17(3):e0264988. doi: 10.1371/journal.pone.0264988 (PMC8946748; doi:10.1371/journal.pone.0264988)
Supplement: S2 Text — (DOCX) [file pone.0264988.s003.docx]

***Sighting Data Citations***

Ampela, K. and C. Bacon. 2018. VACAPES PAX Vessel Opportunistic Sightings July 2015 - Nov 2017. Data downloaded from OBIS-SEAMAP (http://seamap.env.duke.edu/dataset/1815) on 2018-09-18.

Ampela, K. and G. Miller-Francisco. 2016. JAX FIREX Aerial Surveys 5-8 September 2012. Data downloaded from OBIS-SEAMAP (http://seamap.env.duke.edu/dataset/880) on 2019-09-18.

Barco, S. 2013. Virginia Aquarium Marine Mammal Strandings 1988-2008. Data downloaded from OBIS-SEAMAP (http://seamap.env.duke.edu/dataset/502) on 2019-09-18 and 2021-03-04.

Barco, S. 2014. Virginia and Maryland Sea Turtle Research and Conservation Initiative Aerial Survey Sightings, May 2011 through July 2013. Data downloaded from OBIS-SEAMAP (http://seamap.env.duke.edu/dataset/1201) on 2019-09-18.

Barco, S. 2015. Marine Mammal and Sea Turtle Sightings in the Vicinity of the Maryland Wind Energy Area 2013-2015. Data downloaded from OBIS-SEAMAP (http://seamap.env.duke.edu/dataset/1340) on 2019-09-18.

Barco, S. 2015. Virginia CZM Wind Energy Area Survey - Left side - May 2014 through December 2014. Data downloaded from OBIS-SEAMAP (http://seamap.env.duke.edu/dataset/1229) on 2019-09-18.

Barco, S. 2015. Virginia CZM Wind Energy Area Survey - Right side - May 2014 through December 2014. Data downloaded from OBIS-SEAMAP (http://seamap.env.duke.edu/dataset/1231) on 2019-09-18.

Barco, S. 2015. Virginia CZM Wind Energy Area Survey- Right side - November 2012 through April 2014. Data downloaded from OBIS-SEAMAP (http://seamap.env.duke.edu/dataset/1194) on 2019-09-18.

Barco, S. 2016. Virginia CZM Wind Energy Area Survey- Left side - November 2012 through April 2014. Data downloaded from OBIS-SEAMAP (http://seamap.env.duke.edu/dataset/1192) on 2019-09-18.

Boisseau, O. 2014. Visual sightings from Song of the Whale 1993-2013. Data downloaded from OBIS-SEAMAP (http://seamap.env.duke.edu/dataset/1158) on 2019-09-18, 2019-10-11, and 2021-03-04.

Boisseau, O. 2019. Sightings from R/V Song of the Whale during the spring 2019 MAPS survey (NMFS permit 14809). Data downloaded from OBIS-SEAMAP (http://seamap.env.duke.edu/dataset/2001) on 2019-10-18 and 2021-03-04.

Boisseau, O. 2019. Sightings from R/V Song of the Whale during the winter 2019 MAPS survey (NMFS permit 14809). Data downloaded from OBIS-SEAMAP (http://seamap.env.duke.edu/dataset/1999) on 2019-09-18 and 2021-03-04.

Cole, T. and C. Khan. 2016. NEFSC Right Whale Aerial Survey. Data downloaded from OBIS-SEAMAP (http://seamap.env.duke.edu/dataset/513) on 2019-09-18, 2019-10-11, and 2021-03-04.

Contillo, J. 2013. SEFSC Dolphin Photo ID. Data downloaded from OBIS-SEAMAP (http://seamap.env.duke.edu/dataset/226) on 2019-09-18.

Cotter, M. P. 2020. VACAPES NFC Aerial Surveys 2018-2019. Data downloaded from OBIS-SEAMAP (http://seamap.env.duke.edu/dataset/2059) on 2021-03-04.

DFO. (2017). DFO Maritimes Region Cetacean Sightings. Version 7 In OBIS Canada Digital Collections. Bedford Institute of Oceanography, Dartmouth, NS, Canada. Published by OBIS, Digital http://www.iobis.org/. Accessed on 2021-03-04.

Dias, L. and L. Garrison. 2018. AMAPPS Southeast Aerial Cruise Fall 2016. Data downloaded from OBIS-SEAMAP (http://seamap.env.duke.edu/dataset/1854) on 2019-09-18.

Dias, L. and L. Garrison. 2018. AMAPPS Southeast Aerial Cruise Fall 2017. Data downloaded from OBIS-SEAMAP (http://seamap.env.duke.edu/dataset/1858) on 2019-09-18.

Dias, L. and L. Garrison. 2018. AMAPPS Southeast Aerial Cruise Spring 2014. Data downloaded from OBIS-SEAMAP (http://seamap.env.duke.edu/dataset/1850) on 2019-09-18 and 2021-03-04.

Dias, L. and L. Garrison. 2018. AMAPPS Southeast Aerial Cruise Spring 2017. Data downloaded from OBIS-SEAMAP (http://seamap.env.duke.edu/dataset/1860) on 2019-09-18 and 2021-03-04.

Dias, L. and L. Garrison. 2018. AMAPPS Southeast Aerial Cruise Summer 2016. Data downloaded from OBIS-SEAMAP (http://seamap.env.duke.edu/dataset/1856) on 2019-09-18 and 2021-03-04.

Dias, L. and L. Garrison. 2018. AMAPPS Southeast Aerial Cruise Winter 2015. Data downloaded from OBIS-SEAMAP (http://seamap.env.duke.edu/dataset/1852) on 2019-09-18.

Dias, L. and L. Garrison. 2019. AMAPPS Southeast Aerial Cruise Spring 2019. Data downloaded from OBIS-SEAMAP (http://seamap.env.duke.edu/dataset/2036) on 2019-09-18 and 2021-03-04.

Dias, L. and L. Garrison. 2019. AMAPPS Southeast Shipboard Cruise Summer 2016. Data downloaded from OBIS-SEAMAP (http://seamap.env.duke.edu/dataset/1974) on 2019-09-18, 2019-10-11, and 2021-03-04.

Dias, L. and L. Garrison. 2020. AMAPPS Southeast Aerial Cruise Winter 2019-2020. Data downloaded from OBIS-SEAMAP (http://seamap.env.duke.edu/dataset/2053) on 2019-09-18.

Diaz, G. 2011. NOAA Southeast Fishery Science Center (SEFSC) Commercial Pelagic Observer Program (POP) Data. Data downloaded from OBIS-SEAMAP (http://seamap.env.duke.edu/dataset/103151496) on 2019-09-18 and 2021-03-04 and originated from iOBIS (http:www.iobis.org).

DiMatteo, A. 2014. Christopher Newport University bottlenose dolphin sightings in Virginia estuaries 2000-2006. Data downloaded from OBIS-SEAMAP (http://seamap.env.duke.edu/dataset/1074) on 2019-09-18.

Dunn, C. 2006. Bahamas Marine Mammal Research Organisation Aerial Survey. Data downloaded from OBIS-SEAMAP (http://seamap.env.duke.edu/dataset/330) on 2019-09-18 and 2021-03-04.

Dunn, C. 2006. Bahamas Marine Mammal Research Organisation Strandings. Data downloaded from OBIS-SEAMAP (http://seamap.env.duke.edu/dataset/327) on 2019-09-18, 2019-10-11, and 2021-03-04.

Dunn, C. 2013. Bahamas Marine Mammal Research Organisation On-transect Sightings. Data downloaded from OBIS-SEAMAP (http://seamap.env.duke.edu/dataset/328) on 2019-09-18, 2019-10-11, and 2021-03-04.

Dunn, C. 2013. Bahamas Marine Mammal Research Organisation Opportunistic Sightings. Data downloaded from OBIS-SEAMAP (http://seamap.env.duke.edu/dataset/329) on 2019-09-18, 2019-10-11, and 2021-03-04.

Engelhaupt, A. 2019. VACAPES Vessel Nearshore Humpback Whale Monitoring 2015. Data downloaded from OBIS-SEAMAP (http://seamap.env.duke.edu/dataset/2003) on 2019-09-18.

Engelhaupt, A. 2020. VACAPES Offshore Study Area Observations 2015-2018. Data downloaded from OBIS-SEAMAP (http://seamap.env.duke.edu/dataset/2066) on 2019-09-18 and 2021-03-04.

Garrison, L. 2013. SEFSC Atlantic surveys 1992. Data downloaded from OBIS-SEAMAP (http://seamap.env.duke.edu/dataset/3) on 2019-09-18 and 2021-03-04.

Garrison, L. 2013. SEFSC Atlantic surveys 1999. Data downloaded from OBIS-SEAMAP (http://seamap.env.duke.edu/dataset/5) on 2019-09-18 and 2021-03-04.

Garrison, L. 2013. SEFSC Atlantic surveys, 1998 (3). Data downloaded from OBIS-SEAMAP (http://seamap.env.duke.edu/dataset/1) on 2019-09-18 and 2021-03-04.

Garrison, L. 2013. SEFSC Mid-Atlantic Tursiops Survey, 1995 (1). Data downloaded from OBIS-SEAMAP (http://seamap.env.duke.edu/dataset/90) on 2019-09-18.

Garrison, L. 2013. SEFSC Mid-Atlantic Tursiops Survey, 1995 2. Data downloaded from OBIS-SEAMAP (http://seamap.env.duke.edu/dataset/89) on 2019-09-18.

Garrison, L. 2013. SEFSC Mid-Atlantic Tursiops Survey, 1995 3. Data downloaded from OBIS-SEAMAP (http://seamap.env.duke.edu/dataset/88) on 2019-09-18.

Garrison, L. 2013. SEFSC Southeast Cetacean Aerial Survey 1992. Data downloaded from OBIS-SEAMAP (http://seamap.env.duke.edu/dataset/87) on 2019-09-18.

Garrison, L. 2013. SEFSC Southeast Cetacean Aerial Survey 1995. Data downloaded from OBIS-SEAMAP (http://seamap.env.duke.edu/dataset/86) on 2019-09-18 and 2021-03-04.

Gowan, T. 2019. SEUS Right Whale EWS Aerial Surveys 2016-17. Data downloaded from OBIS-SEAMAP (http://seamap.env.duke.edu/dataset/1990) on 2019-09-18.

Gowan, T. 2019. SEUS Right Whale EWS Aerial Surveys 2018-19. Data downloaded from OBIS-SEAMAP (http://seamap.env.duke.edu/dataset/2004) on 2019-09-18.

Gowan, T. and B. Zoodsma. 2019. SEUS Right Whale EWS Aerial Surveys 2017-18. Data downloaded from OBIS-SEAMAP (http://seamap.env.duke.edu/dataset/1992) on 2019-09-18 and 2021-03-04.

Happywhale. 2021. Happywhale - Atlantic White-sided Dolphin in North Atlantic Ocean. Data downloaded from OBIS-SEAMAP (http://seamap.env.duke.edu/dataset/1937) on 2019-09-18and originated from Happywhale.com.

Happywhale. 2021. Happywhale - Common Bottlenose Dolphin in North Atlantic Ocean. Data downloaded from OBIS-SEAMAP (http://seamap.env.duke.edu/dataset/1947) on 2019-09-18 and originated from Happywhale.com.

Harris, Lei E. 2015. DFO Maritimes Region Cetacean Sightings. Version 6 In OBIS Canada Digital Collections. Bedford Institute of Oceanography, Dartmouth, NS, Canada. Published by OBIS, Digital <http://www.iobis.org/>. Data downloaded from OBIS-SEAMAP (http://seamap.env.duke.edu/dataset/103152572) on 2019-09-18, 2019-10-11, and 2021-03-04.

Hartvedt, S. 2020. Incidental sightings of marine mammals. Data downloaded from OBIS-SEAMAP (http://seamap.env.duke.edu/dataset/103152572) on 2021-03-04 and originated from OBIS (https://obis.org/dataset/dd335e79-f580-44a1-bedc-1476437eb73e)

Hyrenbach, D. 2011. Hatteras Eddy Cruise 2004. Data downloaded from OBIS-SEAMAP (http://seamap.env.duke.edu/dataset/322) on 2019-09-18.

Hyrenbach, D. and H. Whitehead. 2008. Sargasso sperm whales 2004. Data downloaded from OBIS-SEAMAP (http://seamap.env.duke.edu/dataset/306) on 2021-03-04.

Hyrenbach, D. and H. Whitehead. 2013. Sargasso 2005 - cetacean sightings. Data downloaded from OBIS-SEAMAP (http://seamap.env.duke.edu/dataset/332) on 2019-09-18 and 2021-03-04.

Hyrenbach, D., F. Huettmann and J. Chardine. 2012. PIROP Northwest Atlantic 1965-1992. Data downloaded from OBIS-SEAMAP (http://seamap.env.duke.edu/dataset/280) on 2019-09-18 and 2021-03-04.

Johnston, D. and Z. Swaim. 2013. DUML vessel-based surveys for proposed JAX USWTR site 2009-2011. Data downloaded from OBIS-SEAMAP (http://seamap.env.duke.edu/dataset/582) on 2019-09-18.

Josephson, B. 2015. AMAPPS Northeast Aerial Cruise Fall 2012. Data downloaded from OBIS-SEAMAP (http://seamap.env.duke.edu/dataset/1245) on 2019-09-18 and 2021-03-04.

Josephson, B. 2015. AMAPPS Northeast Aerial Cruise Spring 2012. Data downloaded from OBIS-SEAMAP (http://seamap.env.duke.edu/dataset/1247) on 2019-09-18 and 2021-03-04.

Josephson, B. 2015. AMAPPS Northeast Aerial Cruise Summer 2010. Data downloaded from OBIS-SEAMAP (http://seamap.env.duke.edu/dataset/1249) on 2019-09-18 and 2021-03-04.

Josephson, B. 2015. AMAPPS Northeast Aerial Cruise Summer 2011. Data downloaded from OBIS-SEAMAP (http://seamap.env.duke.edu/dataset/1233) on 2019-09-18 and 2021-03-04.

Josephson, B. 2015. AMAPPS Northeast Aerial Cruise Winter 2011. Data downloaded from OBIS-SEAMAP (http://seamap.env.duke.edu/dataset/1243) on 2019-09-18.

Josephson, B. 2015. AMAPPS Northeast Shipboard Cruise Summer 2011. Data downloaded from OBIS-SEAMAP (http://seamap.env.duke.edu/dataset/1269) on 2019-09-18, 2019-10-11, and 2021-03-04.

Josephson, B. 2015. AMAPPS Northeast Shipboard Cruise Summer 2013. Data downloaded from OBIS-SEAMAP (http://seamap.env.duke.edu/dataset/1271) on 2019-09-18 and 2021-03-04.

Josephson, B. 2016. AMAPPS Northeast Aerial Cruise Spring 2014. Data downloaded from OBIS-SEAMAP (http://seamap.env.duke.edu/dataset/1379) on 2019-09-18.

Josephson, B. 2016. AMAPPS Northeast Aerial Cruise Winter 2014. Data downloaded from OBIS-SEAMAP (http://seamap.env.duke.edu/dataset/1381) on 2019-09-18.

Josephson, B. 2016. AMAPPS Northeast Shipboard Cruise Spring 2014. Data downloaded from OBIS-SEAMAP (http://seamap.env.duke.edu/dataset/1377) on 2019-09-18 and 2021-03-04.

Josephson, B. 2018. AMAPPS Northeast Aerial Cruise Summer 2016. Data downloaded from OBIS-SEAMAP (http://seamap.env.duke.edu/dataset/1676) on 2019-09-18 and 2021-03-04.

Josephson, B. 2018. AMAPPS Northeast Shipboard Cruise Summer 2016. Data downloaded from OBIS-SEAMAP (http://seamap.env.duke.edu/dataset/1678) on 2019-09-18 and 2021-03-04.

Josephson, B. and D. Palka. 2018. NEFSC Abundance of the Gulf of Maine and Bay of Fundy Harbor Porpoise Based on Aerial Surveys 1999. Data downloaded from OBIS-SEAMAP (http://seamap.env.duke.edu/dataset/1895) on 2019-09-18.

Josephson, B. and D. Palka. 2018. NEFSC Aerial Circle-Back Abundance Survey 2006. Data downloaded from OBIS-SEAMAP (http://seamap.env.duke.edu/dataset/1889) on 2019-09-18 and 2021-03-04.

Josephson, B. and D. Palka. 2018. NEFSC North Atlantic Marine Mammal and Turtle Aerial Abundance Survey 2007. Data downloaded from OBIS-SEAMAP (http://seamap.env.duke.edu/dataset/1891) on 2019-09-18 and 2021-03-04.

Josephson, B. and D. Palka. 2018. NEFSC Twin Otter Aerial Survey 2008. Data downloaded from OBIS-SEAMAP (http://seamap.env.duke.edu/dataset/1893) on 2019-09-18 and 2021-03-04.

Josephson, B. and L. Garrison. 2015. AMAPPS Southeast Aerial Cruise Fall 2012. Data downloaded from OBIS-SEAMAP (http://seamap.env.duke.edu/dataset/1288) on 2019-09-18.

Josephson, B. and L. Garrison. 2015. AMAPPS Southeast Aerial Cruise Spring 2012. Data downloaded from OBIS-SEAMAP (http://seamap.env.duke.edu/dataset/1259) on 2019-09-18 and 2021-03-04.

Josephson, B. and L. Garrison. 2015. AMAPPS Southeast Aerial Cruise Summer 2010. Data downloaded from OBIS-SEAMAP (http://seamap.env.duke.edu/dataset/1273) on 2019-09-18.

Josephson, B. and L. Garrison. 2015. AMAPPS Southeast Aerial Cruise Summer 2011. Data downloaded from OBIS-SEAMAP (http://seamap.env.duke.edu/dataset/1275) on 2019-09-18 and 2021-03-04.

Josephson, B. and L. Garrison. 2015. AMAPPS Southeast Aerial Cruise Winter 2011. Data downloaded from OBIS-SEAMAP (http://seamap.env.duke.edu/dataset/1277) on 2019-09-18 and 2021-03-04.

Josephson, B. and L. Garrison. 2015. AMAPPS Southeast Aerial Cruise Winter 2013. Data downloaded from OBIS-SEAMAP (http://seamap.env.duke.edu/dataset/1289) on 2019-09-18.

Kenney, R. 2013. BLM CETAP AIR Sightings. Data downloaded from OBIS-SEAMAP (http://seamap.env.duke.edu/dataset/283) on 2019-09-18, 2019-10-11, and 2021-03-04.

Kenney, R. 2013. BLM CETAP OPP Sightings. Data downloaded from OBIS-SEAMAP (http://seamap.env.duke.edu/dataset/284) on 2019-09-18, 2019-10-11, and 2021-03-04.

Kenney, R. 2013. BLM CETAP SHIP Sightings. Data downloaded from OBIS-SEAMAP (http://seamap.env.duke.edu/dataset/285) on 2019-09-18, 2019-10-11, and 2021-03-04.

Kopelman, A. 2013. Opportunistic marine mammal sightings from commercial whale watching vessels, Montauk, New York 1981-1994. Data downloaded from OBIS-SEAMAP (http://seamap.env.duke.edu/dataset/1006) on 2019-09-18 and 2021-03-04.

Kopelman, A. 2015. CRESLI marine mammal observations from whale watch cruises 2000-2014. Data downloaded from OBIS-SEAMAP (http://seamap.env.duke.edu/dataset/896) on 2019-09-18.

LaBrecque, E. 2011. Cape Hatteras 04-05. Data downloaded from OBIS-SEAMAP (http://seamap.env.duke.edu/dataset/298) on 2019-09-18 and 2021-03-04.

Lapolla, F. 2013. The Dolphin Project. Data downloaded from OBIS-SEAMAP (http://seamap.env.duke.edu/dataset/304) on 2019-09-18.

Lockhart, G. and S. Barco. 2017. Virginia aerial marine species monitoring 2016 by VAQF - Left side -. Data downloaded from OBIS-SEAMAP (http://seamap.env.duke.edu/dataset/1494) on 2019-09-18.

Lockhart, G. and S. Barco. 2017. Virginia aerial marine species monitoring 2016 by VAQF - Right side -. Data downloaded from OBIS-SEAMAP (http://seamap.env.duke.edu/dataset/1495) on 2019-09-18.

Mallette S.D., Lockhart G G., McAlarney R.J., Cummings E.W., Pabst D. A., McLellan W.A., Barco S.G. 2016. Offshore Energy Planning: Documenting Megafauna off Virginiaâ€™s Coast Using Aerial Surveys. VAQF Scientific Report. 2016-04.

Mallette S.D., Lockhart G G., McAlarney R.J., Cummings E.W., Pabst D. A., McLellan W.A., Barco S.G. 2016. Offshore Energy Planning: Documenting Megafauna off Virginiaâ€™s Coast Using Aerial Surveys. VAQF Scientific Report. 2016-04.

Maughan, B. and K. Arnold. 2010. UK Royal Navy Marine Mammal Observations. Data downloaded from OBIS-SEAMAP (http://seamap.env.duke.edu/dataset/64) on 2019-09-18.

McLellan, W. 2005. UNCW Aerial Survey 1998-1999. Data downloaded from OBIS-SEAMAP (http://seamap.env.duke.edu/dataset/272) on 2019-09-18 and 2021-03-04.

McLellan, W. 2006. UNCW Marine Mammal Sightings 1998-1999. Data downloaded from OBIS-SEAMAP (http://seamap.env.duke.edu/dataset/66) on 2019-09-18 and 2021-03-04.

McLellan, W. 2007. UNCW Marine Mammal Sightings 2002. Data downloaded from OBIS-SEAMAP (http://seamap.env.duke.edu/dataset/67) on 2019-09-18.

McLellan, W. 2010. UNCW Marine Mammal Sightings 2001. Data downloaded from OBIS-SEAMAP (http://seamap.env.duke.edu/dataset/65) on 2019-09-18.

McLellan, W. 2011. UNCW Aerial Surveys for monitoring of proposed Onslow Bay USWTR site - Left side -. Data downloaded from OBIS-SEAMAP (http://seamap.env.duke.edu/dataset/435) on 2019-09-18.

McLellan, W. 2011. UNCW Marine Mammal Aerial Surveys 2006-2007. Data downloaded from OBIS-SEAMAP (http://seamap.env.duke.edu/dataset/400) on 2019-09-18.

McLellan, W. 2011. UNCW Right Whale Aerial Survey 05-06. Data downloaded from OBIS-SEAMAP (http://seamap.env.duke.edu/dataset/360) on 2019-09-18 and 2021-03-04.

McLellan, W. 2011. UNCW USWTR JAX Aerial Surveys May - Oct 2010 - Left side. Data downloaded from OBIS-SEAMAP (http://seamap.env.duke.edu/dataset/687) on 2019-09-18 and 2021-03-04.

McLellan, W. 2011. UNCW USWTR JAX Aerial Surveys May - Oct 2010 - Right side. Data downloaded from OBIS-SEAMAP (http://seamap.env.duke.edu/dataset/688) on 2019-09-18.

McLellan, W. 2011. USWTR JAX Aerial Survey -Left side- 2009-2010. Data downloaded from OBIS-SEAMAP (http://seamap.env.duke.edu/dataset/590) on 2019-09-18.

McLellan, W. 2011. USWTR JAX Aerial Survey -Left side- 2010-2011. Data downloaded from OBIS-SEAMAP (http://seamap.env.duke.edu/dataset/745) on 2019-09-18.

McLellan, W. 2011. USWTR JAX Aerial Survey -Right side- 2010-2011. Data downloaded from OBIS-SEAMAP (http://seamap.env.duke.edu/dataset/747) on 2019-09-18 and 2021-03-04.

McLellan, W. 2011. USWTR Onslow Bay Aerial Survey -Left side- 2008-2010. Data downloaded from OBIS-SEAMAP (http://seamap.env.duke.edu/dataset/586) on 2019-09-18.

McLellan, W. 2011. USWTR Onslow Bay Aerial Survey -Left side- 2010-2011. Data downloaded from OBIS-SEAMAP (http://seamap.env.duke.edu/dataset/749) on 2019-09-18.

McLellan, W. 2011. USWTR Onslow Bay Aerial Survey -Right side- 2008-2010. Data downloaded from OBIS-SEAMAP (http://seamap.env.duke.edu/dataset/588) on 2019-09-18.

McLellan, W. 2011. USWTR Onslow Bay Aerial Survey -Right side- 2010-2011. Data downloaded from OBIS-SEAMAP (http://seamap.env.duke.edu/dataset/751) on 2019-09-18.

McLellan, W. 2012. USWTR JAX Aerial Survey -Left side- 2011-2012. Data downloaded from OBIS-SEAMAP (http://seamap.env.duke.edu/dataset/857) on 2019-09-18.

McLellan, W. 2012. USWTR JAX Aerial Survey -Right side- 2009-2010. Data downloaded from OBIS-SEAMAP (http://seamap.env.duke.edu/dataset/592) on 2019-09-18.

McLellan, W. 2012. USWTR JAX Aerial Survey -Right side- 2011-2012. Data downloaded from OBIS-SEAMAP (http://seamap.env.duke.edu/dataset/859) on 2019-09-18.

McLellan, W. 2013. UNCW Aerial Surveys for monitoring of proposed Onslow Bay USWTR site - Right side -. Data downloaded from OBIS-SEAMAP (http://seamap.env.duke.edu/dataset/437) on 2019-09-18.

McLellan, W. 2013. UNCW Right Whale Aerial Surveys 2008. Data downloaded from OBIS-SEAMAP (http://seamap.env.duke.edu/dataset/464) on 2019-09-18 and 2021-03-04.

McLellan, W. 2014. AFAST Hatteras Aerial Survey -Left side- 2011-2012. Data downloaded from OBIS-SEAMAP (http://seamap.env.duke.edu/dataset/851) on 2019-09-18 and 2021-03-04.

McLellan, W. 2014. AFAST Hatteras Aerial Survey -Right side- 2011-2012. Data downloaded from OBIS-SEAMAP (http://seamap.env.duke.edu/dataset/855) on 2019-09-18 and 2021-03-04.

McLellan, W. 2014. AFTT Hatteras Aerial Survey -Left side- 2012-2013. Data downloaded from OBIS-SEAMAP (http://seamap.env.duke.edu/dataset/1138) on 2019-09-18 and 2021-03-04.

McLellan, W. 2014. AFTT Hatteras Aerial Survey -Right side- 2012-2013. Data downloaded from OBIS-SEAMAP (http://seamap.env.duke.edu/dataset/1140) on 2019-09-18 and 2021-03-04.

McLellan, W. 2014. AFTT JAX Aerial Survey -Left side- 2012-2013. Data downloaded from OBIS-SEAMAP (http://seamap.env.duke.edu/dataset/1128) on 2019-09-18.

McLellan, W. 2014. AFTT JAX Aerial Survey -Right side- 2012-2013. Data downloaded from OBIS-SEAMAP (http://seamap.env.duke.edu/dataset/1136) on 2019-09-18.

McLellan, W. 2015. AFTT Cape Hatteras Aerial Survey -Left side- 2014. Data downloaded from OBIS-SEAMAP (http://seamap.env.duke.edu/dataset/1237) on 2019-09-18 and 2021-03-04.

McLellan, W. 2015. AFTT Cape Hatteras Aerial Survey -Right side- 2014. Data downloaded from OBIS-SEAMAP (http://seamap.env.duke.edu/dataset/1235) on 2019-09-18 and 2021-03-04.

McLellan, W. 2015. AFTT JAX Aerial Survey -Left side- 2014. Data downloaded from OBIS-SEAMAP (http://seamap.env.duke.edu/dataset/1241) on 2019-09-18.

McLellan, W. 2015. AFTT JAX Aerial Survey -Right side- 2014. Data downloaded from OBIS-SEAMAP (http://seamap.env.duke.edu/dataset/1239) on 2019-09-18 and 2021-03-04.

McLellan, W. 2016. UNCW Hatteras Aerial Survey - Left side - 2015. Data downloaded from OBIS-SEAMAP (http://seamap.env.duke.edu/dataset/1350) on 2019-09-18 and 2021-03-04.

McLellan, W. 2016. UNCW Hatteras Aerial Survey - Right side - 2015. Data downloaded from OBIS-SEAMAP (http://seamap.env.duke.edu/dataset/1352) on 2019-09-18 and 2021-03-04.

McLellan, W. 2016. UNCW JAX Aerial Survey - Left side - 2015. Data downloaded from OBIS-SEAMAP (http://seamap.env.duke.edu/dataset/1362) on 2019-09-18.

McLellan, W. 2016. UNCW JAX Aerial Survey - Right side - 2015. Data downloaded from OBIS-SEAMAP (http://seamap.env.duke.edu/dataset/1364) on 2019-09-18.

McLellan, W. 2016. UNCW Norfolk Canyon Aerial Survey - Left side - 2015. Data downloaded from OBIS-SEAMAP (http://seamap.env.duke.edu/dataset/1354) on 2019-09-18 and 2021-03-04.

McLellan, W. 2016. UNCW Norfolk Canyon Aerial Survey - Right side - 2015. Data downloaded from OBIS-SEAMAP (http://seamap.env.duke.edu/dataset/1356) on 2019-09-18 and 2021-03-04.

McLellan, W. 2016. UNCW PAX Aerial Survey - Left side - 2015. Data downloaded from OBIS-SEAMAP (http://seamap.env.duke.edu/dataset/1358) on 2019-09-18.

McLellan, W. 2016. UNCW PAX Aerial Survey - Right side - 2015. Data downloaded from OBIS-SEAMAP (http://seamap.env.duke.edu/dataset/1360) on 2019-09-18.

McLellan, W. 2017. UNCW Hatteras Aerial Survey - Left side - 2016. Data downloaded from OBIS-SEAMAP (http://seamap.env.duke.edu/dataset/1471) on 2019-09-18 and 2021-03-04.

McLellan, W. 2017. UNCW Hatteras Aerial Survey - Right side - 2016. Data downloaded from OBIS-SEAMAP (http://seamap.env.duke.edu/dataset/1473) on 2019-09-18 and 2021-03-04.

McLellan, W. 2017. UNCW JAX Aerial Survey - Left side - 2016. Data downloaded from OBIS-SEAMAP (http://seamap.env.duke.edu/dataset/1475) on 2019-09-18.

McLellan, W. 2017. UNCW JAX Aerial Survey - Right side - 2016. Data downloaded from OBIS-SEAMAP (http://seamap.env.duke.edu/dataset/1477) on 2019-09-18.

McLellan, W. 2017. UNCW Norfolk Canyon Aerial Survey - Left side - 2016. Data downloaded from OBIS-SEAMAP (http://seamap.env.duke.edu/dataset/1479) on 2019-09-18 and 2021-03-04.

McLellan, W. 2017. UNCW Norfolk Canyon Aerial Survey - Right side - 2016. Data downloaded from OBIS-SEAMAP (http://seamap.env.duke.edu/dataset/1481) on 2019-09-18 and 2021-03-04.

McLellan, W. 2017. UNCW PAX Aerial Survey - Left side - 2017. Data downloaded from OBIS-SEAMAP (http://seamap.env.duke.edu/dataset/1652) on 2019-09-18.

McLellan, W. 2017. UNCW PAX Aerial Survey - Right side - 2017. Data downloaded from OBIS-SEAMAP (http://seamap.env.duke.edu/dataset/1654) on 2019-09-18.

McLellan, W. 2018. UNCW Hatteras Aerial Survey -Left side- 2017. Data downloaded from OBIS-SEAMAP (http://seamap.env.duke.edu/dataset/1668) on 2019-09-18 and 2021-03-04.

McLellan, W. 2018. UNCW Hatteras Aerial Survey -Right side- 2017. Data downloaded from OBIS-SEAMAP (http://seamap.env.duke.edu/dataset/1670) on 2019-09-18 and 2021-03-04.

McLellan, W. 2018. UNCW JAX Aerial Survey -Left side- 2017. Data downloaded from OBIS-SEAMAP (http://seamap.env.duke.edu/dataset/1662) on 2019-09-18 and 2021-03-04.

McLellan, W. 2018. UNCW JAX Aerial Survey -Right side- 2017. Data downloaded from OBIS-SEAMAP (http://seamap.env.duke.edu/dataset/1664) on 2019-09-18.

McLellan, W. 2018. UNCW Norfolk Canyon Aerial Survey -Left side- 2017. Data downloaded from OBIS-SEAMAP (http://seamap.env.duke.edu/dataset/1672) on 2019-09-18 and 2021-03-04.

McLellan, W. 2018. UNCW Norfolk Canyon Aerial Survey -Right side- 2017. Data downloaded from OBIS-SEAMAP (http://seamap.env.duke.edu/dataset/1674) on 2019-09-18 and 2021-03-04.

McLellan, W. 2018. UNCW PAX Aerial Survey - Left side - 2016. Data downloaded from OBIS-SEAMAP (http://seamap.env.duke.edu/dataset/1797) on 2019-09-18.

McLellan, W. 2018. UNCW PAX Aerial Survey - Right side - 2016. Data downloaded from OBIS-SEAMAP (http://seamap.env.duke.edu/dataset/1799) on 2019-09-18.

NEFSC. 2018. 2017 Annual Report of a Comprehensive Assessment of Marine Mammal, Marine Turtle, and Seabird Abundance and Spatial Distribution in US waters of the Western North Atlantic Ocean – AMAPPS II. Annual Report. Available at: https://www.fisheries.noaa.gov/resource/publication-database/atlantic-marine-assessment-program-protected-species.

NEFSC. 2020. 2019 Annual Report of a Comprehensive Assessment of Marine Mammal, Marine Turtle, and Seabird Abundance and Spatial Distribution in US waters of the Western North Atlantic Ocean – AMAPPS II. Annual Report. TO BE PUBLISHED.

NEFSC. 2020. 2019 Annual Report of a Comprehensive Assessment of Marine Mammal, Marine Turtle, and Seabird Abundance and Spatial Distribution in US waters of the Western North Atlantic Ocean – AMAPPS II. Annual Report. TO BE PUBLISHED.

Normandeau Associates and APEM data prepared for New York State Energy Research and Development Authority. 2019. Digital Aerial Baseline Survey of Marine Wildlife in Support of Offshore Wind Energy. NYSERDA.

Osman, R. 2011. A Biological Survey of the Waters of Woods Hole and Vacinity. Data downloaded from OBIS-SEAMAP (http://seamap.env.duke.edu/dataset/103150230) on 2019-10-11 and originated from iOBIS (http:www.iobis.org).

Palka, D. 2011. NEFSC 1995 AJ9501 (Part I). Data downloaded from OBIS-SEAMAP (http://seamap.env.duke.edu/dataset/56) on 2019-09-18 and 2021-03-04.

Palka, D. 2013. Harbor Porpoise Survey 1992 (AJ92-01). Data downloaded from OBIS-SEAMAP (http://seamap.env.duke.edu/dataset/302) on 2019-09-18.

Palka, D. 2013. NEFSC 1995 AJ9501 (Part II). Data downloaded from OBIS-SEAMAP (http://seamap.env.duke.edu/dataset/290) on 2019-09-18.

Palka, D. 2013. NEFSC 1995 pe9501. Data downloaded from OBIS-SEAMAP (http://seamap.env.duke.edu/dataset/296) on 2019-09-18 and 2021-03-04.

Palka, D. 2013. NEFSC 1995 pe9502. Data downloaded from OBIS-SEAMAP (http://seamap.env.duke.edu/dataset/294) on 2019-09-18 and 2021-03-04.

Palka, D. 2013. NEFSC 1999 aj9902. Data downloaded from OBIS-SEAMAP (http://seamap.env.duke.edu/dataset/300) on 2019-09-18 and 2019-10-11.

Palka, D. 2013. NEFSC Aerial Circle-Back Abundance Survey 2004. Data downloaded from OBIS-SEAMAP (http://seamap.env.duke.edu/dataset/398) on 2019-09-18.

Palka, D. 2013. NEFSC Aerial Survey - Experimental 2002. Data downloaded from OBIS-SEAMAP (http://seamap.env.duke.edu/dataset/107) on 2019-09-18 and 2021-03-04.

Palka, D. 2013. NEFSC Aerial Survey - Summer 1995. Data downloaded from OBIS-SEAMAP (http://seamap.env.duke.edu/dataset/109) on 2019-09-18 and 2021-03-04.

Palka, D. 2013. NEFSC Aerial Survey - Summer 1998. Data downloaded from OBIS-SEAMAP (http://seamap.env.duke.edu/dataset/113) on 2019-09-18 and 2021-03-04.

Palka, D. 2013. NEFSC Deepwater Marine Mammal 2002. Data downloaded from OBIS-SEAMAP (http://seamap.env.duke.edu/dataset/292) on 2019-09-18 and 2021-03-04.

Palka, D. 2013. NEFSC Harbor Porpoise 1991. Data downloaded from OBIS-SEAMAP (http://seamap.env.duke.edu/dataset/288) on 2019-09-18 and 2021-03-04.

Palka, D. 2013. NEFSC Mid-Atlantic Marine Mammal Abundance Survey 2004. Data downloaded from OBIS-SEAMAP (http://seamap.env.duke.edu/dataset/396) on 2019-09-18 and 2021-03-04.

Palka, D. 2013. NEFSC Survey 1997. Data downloaded from OBIS-SEAMAP (http://seamap.env.duke.edu/dataset/58) on 2019-09-18 and 2021-03-04.

Palka, D. 2013. NEFSC Survey 1998 1. Data downloaded from OBIS-SEAMAP (http://seamap.env.duke.edu/dataset/60) on 2019-09-18 and 2021-03-04.

Palka, D. 2013. NEFSC Survey 1998 2. Data downloaded from OBIS-SEAMAP (http://seamap.env.duke.edu/dataset/62) on 2019-09-18 and 2021-03-04.

Palka, D. 2014. NEFSC Survey 1991. Data downloaded from OBIS-SEAMAP (http://seamap.env.duke.edu/dataset/111) on 2019-09-18.

Rappucci, G. and L. Garrison. 2019. SEFSC GoMMAPPS 2017 Summer Aerial Survey. Data downloaded from OBIS-SEAMAP (http://seamap.env.duke.edu/dataset/1970) on 2019-09-18.

Rappucci, G. and L. Garrison. 2019. SEFSC GoMMAPPS 2018 Fall Aerial Survey. Data downloaded from OBIS-SEAMAP (http://seamap.env.duke.edu/dataset/1972) on 2019-09-18.

Read, A. 2011. Duke Cherry Point PopUps 2005-2006 Bottlenose dolphin whistle presence. Data downloaded from OBIS-SEAMAP (http://seamap.env.duke.edu/dataset/567) on 2019-09-18.

Smith, A. 2014. Mystic Aquarium's marine mammal and sea turtle stranding data 1976-2011. Data downloaded from OBIS-SEAMAP (http://seamap.env.duke.edu/dataset/945) on 2019-09-18 and 2021-03-04.

Speakman, T. 2011. NOAA Atlantic bottlenose dolphin sightings in the coastal and estuarine waters near Charleston, SC - 1994-2011. Data downloaded from OBIS-SEAMAP (http://seamap.env.duke.edu/dataset/737) on 2019-09-18.

Spontak, D. 2012. JAX ASWEX Aerial Monitoring 2011. Data downloaded from OBIS-SEAMAP (http://seamap.env.duke.edu/dataset/868) on 2019-09-18.

Spontak, D. 2012. JAX MAVEX Aerial Monitoring 2012. Data downloaded from OBIS-SEAMAP (http://seamap.env.duke.edu/dataset/875) on 2019-09-18.

Spontak, D. 2012. JAX MISSILEX Aerial Monitoring 2010. Data downloaded from OBIS-SEAMAP (http://seamap.env.duke.edu/dataset/874) on 2019-09-18.

Spontak, D. 2012. JAX SEASWITI Aerial Monitoring 2010 . Data downloaded from OBIS-SEAMAP (http://seamap.env.duke.edu/dataset/866) on 2019-09-18.

Spontak, D. 2012. JAX SEASWITI Vessel Monitoring 2010. Data downloaded from OBIS-SEAMAP (http://seamap.env.duke.edu/dataset/867) on 2019-09-18.

Spontak, D. 2012. VACAPES ASWEX Aerial Monitoring 2011. Data downloaded from OBIS-SEAMAP (http://seamap.env.duke.edu/dataset/869) on 2019-09-18.

Spontak, D. 2012. VACAPES FIREX Aerial Monitoring 2011. Data downloaded from OBIS-SEAMAP (http://seamap.env.duke.edu/dataset/871) on 2019-09-18.

Spontak, D. 2013. JAX GUNEX Aerial Monitoring Surveys October 2010. Data downloaded from OBIS-SEAMAP (http://seamap.env.duke.edu/dataset/893) on 2019-09-18.

Spontak, D. 2013. JAX MAVEX September 2012. Data downloaded from OBIS-SEAMAP (http://seamap.env.duke.edu/dataset/895) on 2019-09-18.

Spontak, D. 2013. NSN Monitoring for Pier 1 Upgrades 2011-2012. Data downloaded from OBIS-SEAMAP (http://seamap.env.duke.edu/dataset/894) on 2019-09-18.

Spontak, D. 2013. VACAPES FIREX and ASW Aerial Monitoring 2010. Data downloaded from OBIS-SEAMAP (http://seamap.env.duke.edu/dataset/870) on 2019-09-18.

Spontak, D. 2013. VACAPES MISSELEX Aerial Monitoring March 2013. Data downloaded from OBIS-SEAMAP (http://seamap.env.duke.edu/dataset/1017) on 2019-09-18.

Spontak, D. 2014. Norfolk/VA Beach MINEX Vessel Surveys. Data downloaded from OBIS-SEAMAP (http://seamap.env.duke.edu/dataset/1072) on 2019-09-18.

Spontak, D. 2014. Norfolk/VA Beach Photo-ID Surveys Aug 2012-Sep 2013. Data downloaded from OBIS-SEAMAP (http://seamap.env.duke.edu/dataset/1166) on 2019-09-18.

Spontak, D. 2015. Norfolk/VA Beach Inshore Vessel Surveys Nov 2012- Nov 2013. Data downloaded from OBIS-SEAMAP (http://seamap.env.duke.edu/dataset/1071) on 2019-09-18.

Swaim, Z. 2016. DUML vessel-based photo-id and biopsy surveys for proposed JAX USWTR site 2012-2015. Data downloaded from OBIS-SEAMAP (http://seamap.env.duke.edu/dataset/906) on 2019-09-18.

Swaim, Z. 2016. DUML vessel-based photo-id and biopsy surveys in Onslow Bay CHPT OPAREA 2011-2015. Data downloaded from OBIS-SEAMAP (http://seamap.env.duke.edu/dataset/902) on 2019-09-18.

Swaim, Z. 2016. DUML vessel-based photo-id and biopsy surveys in VACAPES OPAREA off Hatteras 2009, 2011-2015. Data downloaded from OBIS-SEAMAP (http://seamap.env.duke.edu/dataset/907) on 2019-09-18 and 2021-03-04.

Taylor, J. 2015. Bottlenose dolphins off Outer Banks 2007-2012. Data downloaded from OBIS-SEAMAP (http://seamap.env.duke.edu/dataset/837) on 2019-09-18.

Urian, K. 2013. DUML New River surveys on the occurrence, distribution and density of marine mammals in Camp Lejeune 2010-2011. Data downloaded from OBIS-SEAMAP (http://seamap.env.duke.edu/dataset/959) on 2019-09-18.

Urian, K. 2013. DUML surveys for the stock discrimination of bottlenose dolphins along the Outer Banks of North Carolina 2011-2012. Data downloaded from OBIS-SEAMAP (http://seamap.env.duke.edu/dataset/1010) on 2019-09-18.

Urian, K. 2013. DUML vessel-based line transect surveys for proposed Onslow Bay USWTR site 2007-2010. Data downloaded from OBIS-SEAMAP (http://seamap.env.duke.edu/dataset/433) on 2019-09-18.

Urian, K. 2014. DUML coastal surveys on the occurrence, distribution and density of marine mammals in Camp Lejeune 2010-2013. Data downloaded from OBIS-SEAMAP (http://seamap.env.duke.edu/dataset/957) on 2019-09-18.

Vukovich, M. 2018. Digital Aerial Baseline Survey of Marine Wildlife in Support of Offshore Wind Energy - OPA 2016. Data downloaded from OBIS-SEAMAP (http://seamap.env.duke.edu/dataset/1817) on 2019-09-18 and 2021-03-04.

Vukovich, M. 2019. Digital Aerial Baseline Survey of Marine Wildlife in Support of Offshore Wind Energy - OPA 2017. Data downloaded from OBIS-SEAMAP (http://seamap.env.duke.edu/dataset/1994) on 2019-09-18 and 2021-03-04.

Whitt, A. 2015. Marine mammal records of Cuba. Data downloaded from OBIS-SEAMAP (http://seamap.env.duke.edu/dataset/1190) on 2019-10-11.

Wolff, N. 2011. Aerial survey of upper trophic level predators on PLatts Bank, Gulf of Maine. Data downloaded from OBIS-SEAMAP (http://seamap.env.duke.edu/dataset/103150267) on 2019-09-18 and originated from iOBIS (http:www.iobis.org).

Woolmer, G. 2013. Historical distribution of whales shown by logbook records 1785-1913. Data downloaded from OBIS-SEAMAP (http://seamap.env.duke.edu/dataset/885) on 2021-03-04.
