# Supplementary figures and images for: Identification of western North Atlantic odontocete echolocation click types using machine learning and spatiotemporal correlates
Source: PLoS One. 2022 Mar 24;17(3):e0264988. doi: 10.1371/journal.pone.0264988 (PMC8946748; doi:10.1371/journal.pone.0264988)

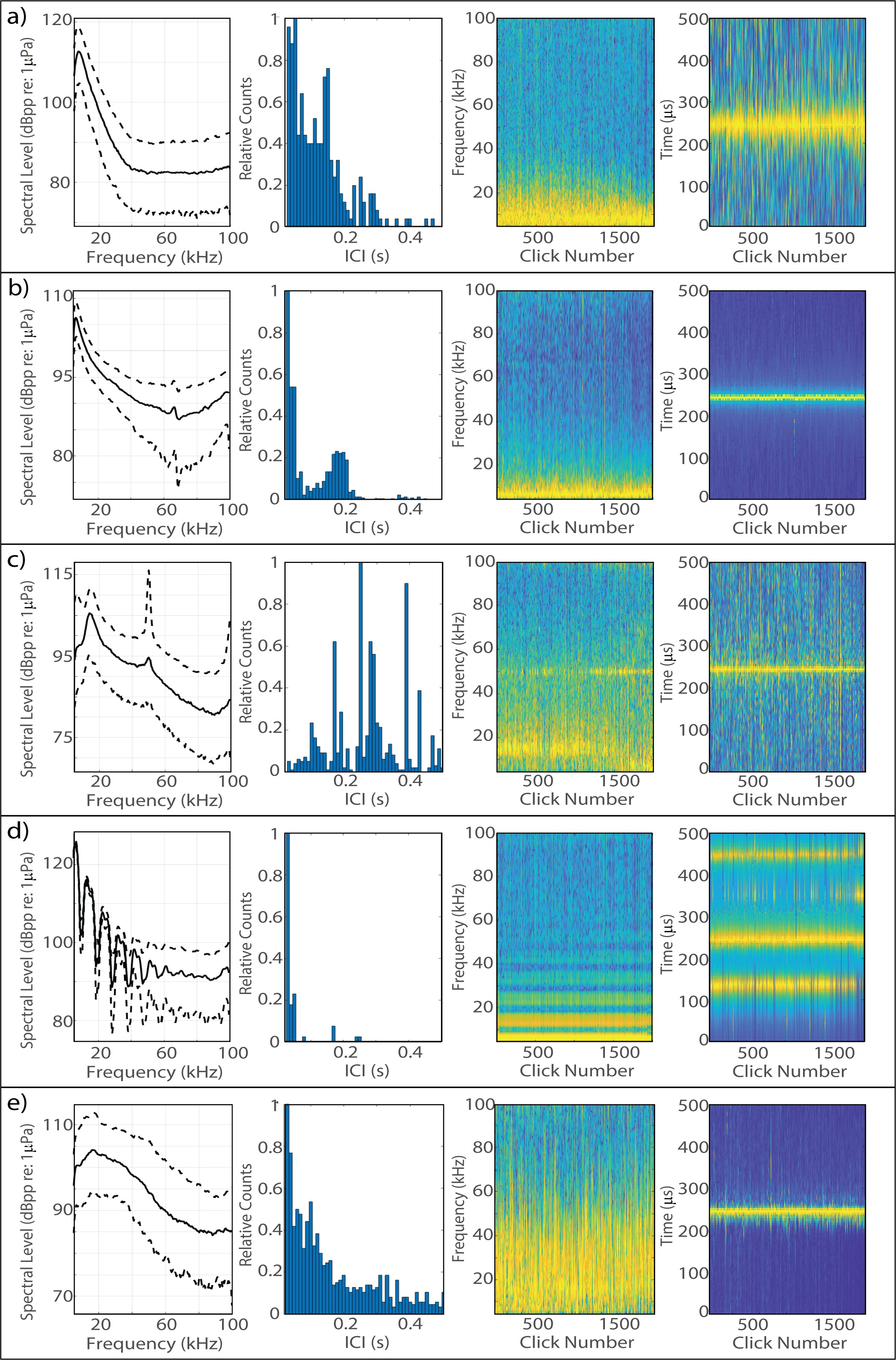

Supplement: S1 Fig — Columns are: median power spectrum (solid line) with 10th and 90th percentiles (dashed lines); distribution of modal IPI values from 1000 5-minute bins; concatenation of normalized impulsive signal spectra, sorted by received level; concatenation of normalized waveform envelopes, sorted by received level. For the concatenated spectra and waveform envelopes, the normalized magnitude of the frequency/pressure is represented by color such that warmer colors show greater magnitude. (TIF) [file pone.0264988.s004.tif]

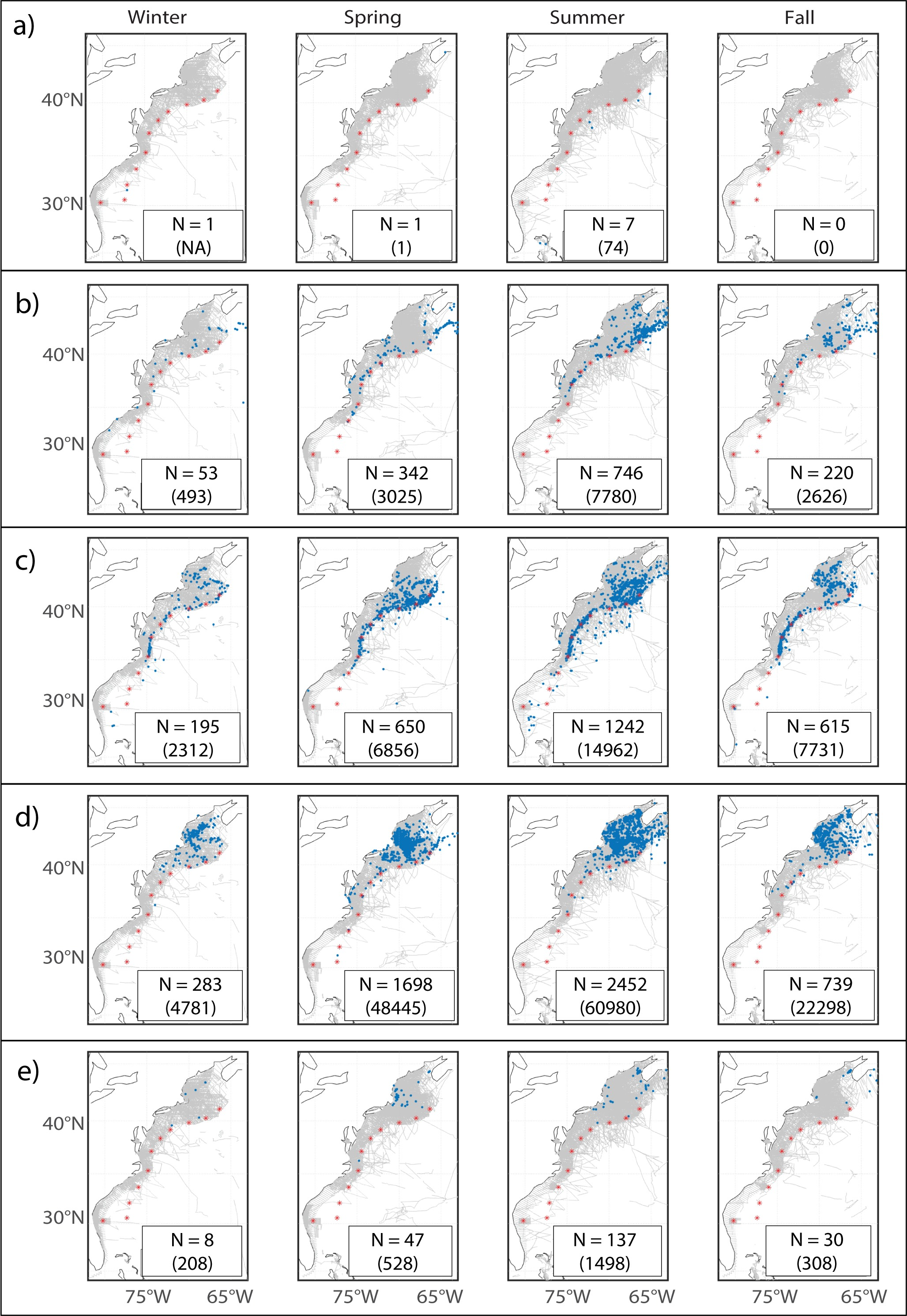

Supplement: S2 Fig — Sightings are plotted per season (blue dots), shown relative to acoustic monitoring sites (red stars) and track lines of surveys undertaken in each season (grey lines). Inset within each sighting map shows number of sightings; total number of individuals summed across all sightings for which group size data was available is given in parentheses. (TIF) [file pone.0264988.s005.tif]

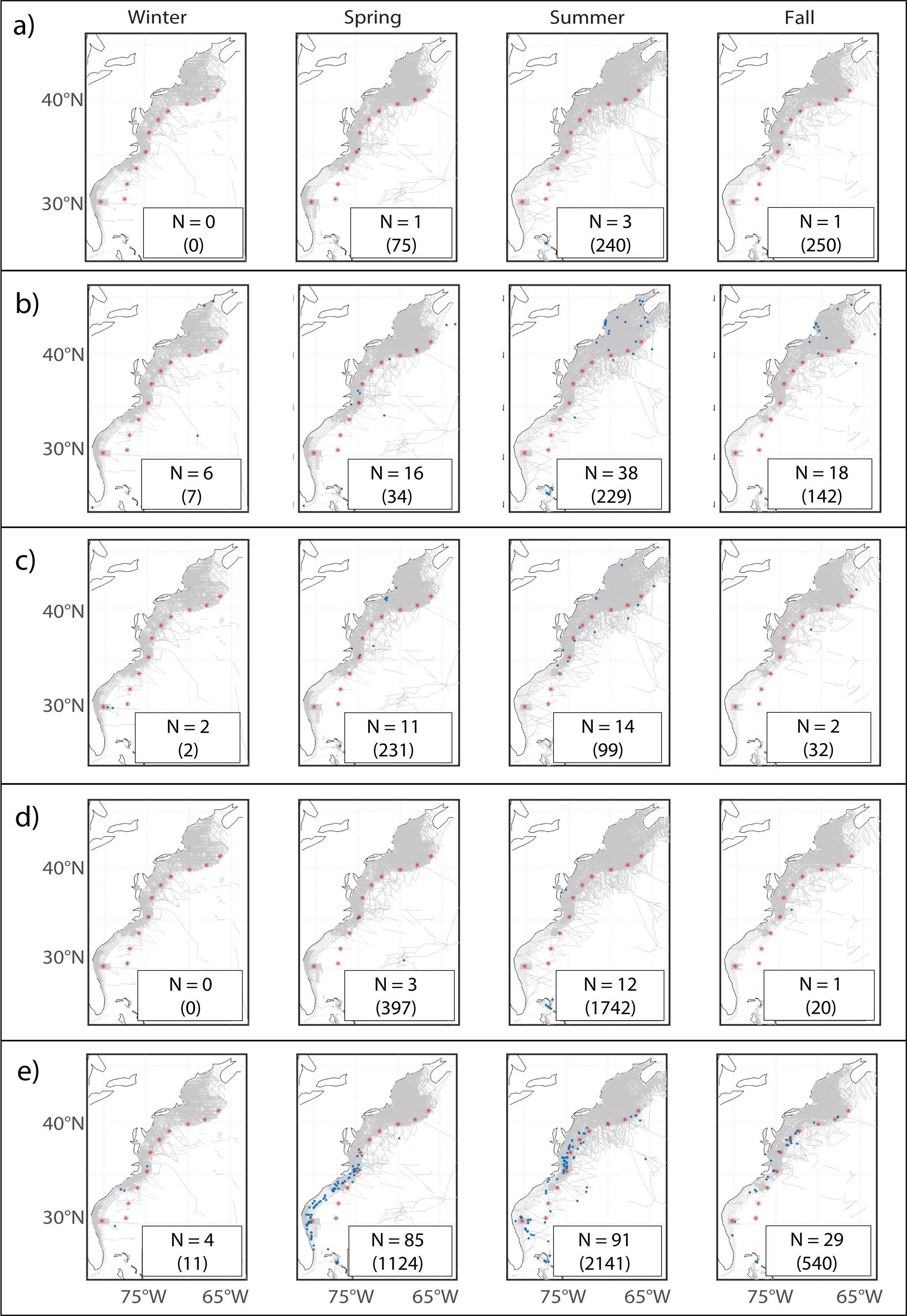

Supplement: S3 Fig — Sightings are plotted per season (blue dots), shown relative to acoustic monitoring sites (red stars) and track lines of surveys undertaken in each season (grey lines). Inset within each sighting map shows number of sightings; total number of individuals summed across all sightings for which group size data was available is given in parentheses. (TIF) [file pone.0264988.s006.tif]

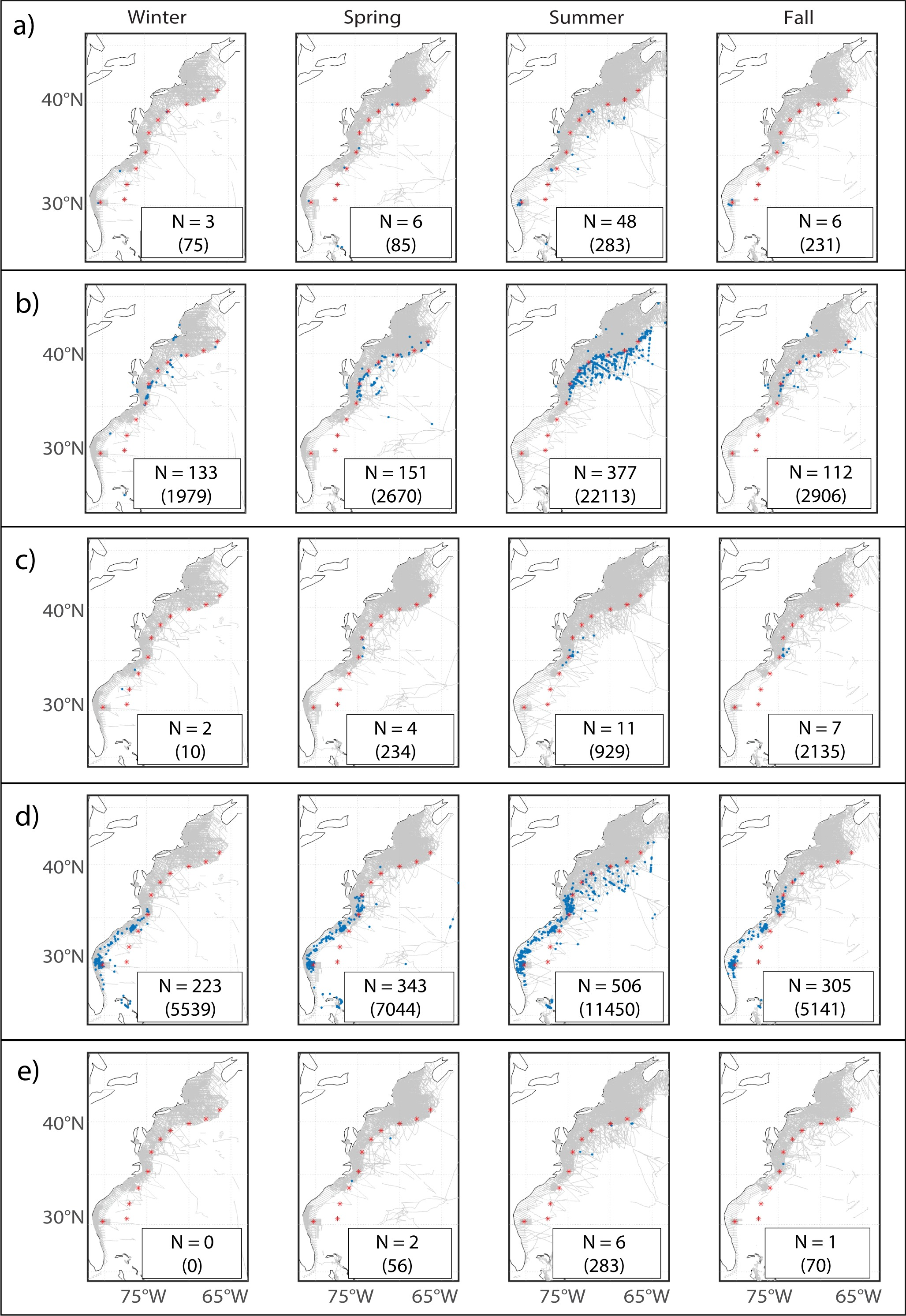

Supplement: S4 Fig — Sightings are plotted per season (blue dots), shown relative to acoustic monitoring sites (red stars) and track lines of surveys undertaken in each season (grey lines). Inset within each sighting map shows number of sightings; total number of individuals summed across all sightings for which group size data was available is given in parentheses. (TIF) [file pone.0264988.s007.tif]

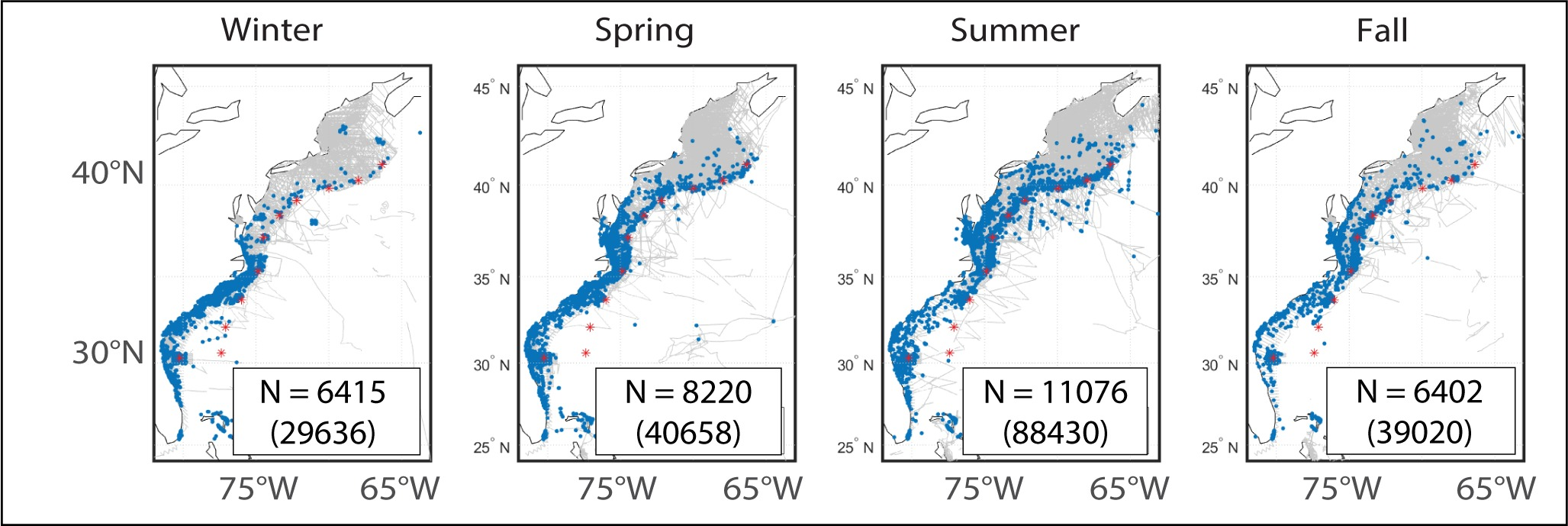

Supplement: S5 Fig — Sightings are plotted per season (blue dots), shown relative to acoustic monitoring sites (red stars) and track lines of surveys undertaken in each season (grey lines). Inset within each sighting map shows number of sightings; total number of individuals summed across all sightings for which group size data was available is given in parentheses. (TIF) [file pone.0264988.s008.tif]
